# Estimation of glucose rate of appearance in portal vein circulation using a phenomenological-based model

**DOI:** 10.1371/journal.pone.0285849

**Published:** 2023-05-25

**Authors:** Laura Lema-Perez, Alejandro Herrón-Bedoya, Valentina Paredes-Ángel, Andrea Hernández-Arango, Carlos E. Builes-Montaño, Hernan Alvarez

**Affiliations:** 1 Artificial Pancreas Trondheim (APT), Norwegian University of Science and Technology (NTNU), Trondheim, Norway; 2 Kalman research group, Facultad de Minas, Universidad Nacional de Colombia, Medellín, Colombia; 3 Hospital Pablo Tobón Uribe and Universidad de Antioquia, Medellín, Colombia; University of Illinois, UNITED STATES

## Abstract

The joint work of the stomach and the small intestine plays a fundamental role in human digestion. In the stomach, food is turned into a semi-fluid mixture that is slowly released into the small intestine, where most enzymatic reactions occur, and nutrients are absorbed as they become available. This whole process is closely related to glucose homeostasis, mainly because of the appearance of glucose in the portal system and the energetic expenditure of the process itself. The current phenomenological-based model describes such effects of the digestive process on blood glucose concentration. It considers enzymatic and mechanical transformations, energetic expenditure, and the impact of macro-nutrients, fiber, and water on overall digestion and glucose absorption. The model estimates the rate of glucose appearance in the portal vein and is intended to be further integrated into existing models for other human organs and used in model-based systems such as an artificial pancreas with automated insulin delivery.

## Introduction

Glucose homeostasis is a process by which glucose levels remain stable overall, even though glucose molecules constantly appear and disappear from the bloodstream as they are absorbed, generated, and consumed in different tissues. The gastrointestinal system plays a significant role in such equilibrium since it processes ingested food, leading to the absorption of new glucose molecules and a concurrent energetic expenditure by the mechanical work of the muscles responsible for the peristalsis. In such a process, the ingestion of carbohydrates, fats, protein, and fiber plays a significant role in the rate of glucose appearance in the portal system circulation. The presence of these nutrients changes both the caloric content and the rheological properties of the food mixture, and therefore has a direct impact on the gastric emptying and the glucose absorption rates. Hence, developing a mathematical model to describe how the gastrointestinal system is involved in the postprandial changes in blood glucose concentration becomes an essential task in the management and treatment of diabetes mellitus.

Most physiological models describing blood glucose changes are based on the minimal model. The minimal model and extensions were developed to analyze a venous stimulus delivered as a bolus or continuous infusion [1–3]. However, the experiments required to provide data for these models are cumbersome, take time, expose experimental subjects to uncomfortable situations, and are expensive. Part of these difficulties can be solved if a food stimulus is used instead of an injection. In addition, models that adequately represent the gastrointestinal kinetics of glucose can be helpful when designing control algorithms used in everyday situations by patients. Some of the available gastrointestinal glucose absorption models use a compartment structure to describe gastric emptying using mathematical functions and linear kinetics to describe intestinal absorption [4, 5]. Others had changed the kinetics of gastric emptying and glucose absorption in the intestine to a non-linear model in which gastric emptying is determined by gastric glucose content [6–8]. Finally, some of the most recent models include the dynamics of the effects of insulin and incretin [9] by using a multi-level model that merges both whole body and cellular levels, as well as exogenous insulin [10].

There is a clear need for a mathematical model that considers the physiological aspects involved in human gastrointestinal digestion and nutrient absorption. This article presents an integrated phenomenological-based model for the role of the stomach [11] and small intestine [12] in human glucose homeostasis, constructed by unifying two existing but independent models for the roles of each one of these organs separately. Both models were slightly modified to be coupled, additional physiological aspects were considered in each one, thus obtaining the model of the gastrointestinal tract presented here. However, the model considers the energy expenditure required for peristaltic movements and the effect of water, fats, proteins, and fiber on digestion. Furthermore, a calorie-constant emptying rate is proposed for the stomach, and a new modeling hypothesis is presented for the small intestine portrayed as a series of elastic tanks. The model also includes changes in the volume of the stomach through the digestive process, as well as new kinetic expressions for intestinal absorption and the digestion of nutrients. It is based on first principles such as mass and energy balances. However, it was adjusted using experimental data reported in the literature, rendering a not entirely physiological model while guaranteeing parameter interpretability [13].

## Construction of a PBSM for the role of the stomach and the small intestine in human glucose homeostasis

A phenomenological-based semi-physical model (PBSM) is a specific type of gray-box model in which the basic structure arises from first principles, such as mass and energy balances, and at least one parameter is adjusted using experimental data. Because of its nature, this kind of model has a modular structure and parameter interpretability. This section presents a PBSM to represent the role of the gastrointestinal tract in glucose homeostasis in humans. The model was constructed following a ten-step methodology proposed in [14, 15].

### Pre-construction of the model

The pre-construction of the model consists of all the steps that precede the establishment of the model structure from mass and energy balances.

#### Process description and objective of the model

As ingested food reaches the stomach through the esophagus, the stomach walls start to widen, which leads to the release of gastric juices, mainly composed of water, hydrochloric acid, and pepsin. Mechanical digestion begins in the stomach, and food is propelled against the stomach walls to be mixed. These movements are caused by peristalsis, which implies mechanical work and energy expenditure. Simultaneously, enzymatic digestion of about 5% of the ingested proteins, and 10–30% of the lipids takes place, and lingual enzymes progressively turn these into amino acids, peptides, free fatty acids, and acylglycerols [16].

As the digestive process progresses, more gastric acid is added to the mixture, which, coupled with the appearance of the products of the enzymatic digestion, leads to changes in the viscosity and particle size of the mix, also called chyme. During the *lag* phase of gastric emptying, chyme is processed without emptying until it reaches the appropriate rheological properties to pass to the small intestine. The duration of this stage depends highly on the caloric content, and the amount of lipids and liquids in the meal [17, 18]. Food passes to the small intestine through the pylorus, in a controlled process in which the rate of calories entering the small intestine is kept approximately constant at 1–4 kcal/min [19]. As food begins to leave the stomach, it progressively shrinks and eventually collapses when left empty.

The small intestine is a tubular organ with a length of approximately 6 m, composed of three sub-sections: Duodenum, jejunum, and ileum. Its inner radial layer, the mucosa, is made of epithelial tissue that mediates the transport of substances from the intestinal lumen to the blood and lymph. As chyme enters the small intestine, it is mixed with pancreatic secretions and bile from the liver. Pancreatic secretions contain the enzymes to degrade carbohydrates, proteins and lipids into monomers and oligomers that can be absorbed through the intestinal wall. In contrast, the bile contains the salts needed to emulsify the lipids. Food travels along the intestine due to peristaltic movements, and simultaneously, nutrients are digested and absorbed together with water and electrolytes. Dietary fiber is neither digested nor absorbed in the small intestine, but it affects the properties of the mixture and, therefore, the absorption rate of glucose.

The model objective is to estimate the glucose absorption into the portal system from a mixed meal. In this regard, the question to be solved by the proposed model is: What is the rate of glucose appearance in the bloodstream due to food digestion and absorption in the stomach and small intestine?

#### Modeling hypothesis and level of detail

The model’s level of detail is considered to be macroscopic, since the stomach and the small intestine are independently seen as a whole. A diagram for the modelling hypothesis used for the stomach can be found in [11]. Since the food mixing in the stomach implies energy expenditure, it is modeled using an analogy with the energy losses by friction when a heterogeneous mixture flows through a closed assembly of circular pipe. The procedure followed to determine the length of each pipeline section is broadly described in [11]. The fluid is considered to be driven through the pipe circuit by a pump, which represents the work done by the stomach wall. Thus, the amount of energy that is effectively transferred to the fluid is taken as 50% of the total power of the pump, assuming that half of the energy provided is used by the cells of the stomach wall for peristalsis. As an additional assumption, the pipe circuit is considered to be full of chyme, even though the real scenario is that both chyme and air are inside the stomach. To compensate for this assumption, the friction factor is adjusted from data, and the speed of the meal inside the pipe circuit is considered to be constant, although the cross-sectional area is accordingly widened and reduced to represent gastric dilution and emptying, respectively. It is assumed that the tissue forming the stomach wall does not retain or lose mass.

As the process begins, there is an inflow of gastric juice to the stomach. Since the distension of the stomach walls is directly related to the release of gastric juices, this flow is considered proportional to the stomach volume at every moment. The small intestine’s neural and hormonal mechanisms provoke feedback control over gastric emptying, and as chyme reaches the duodenum, gastric secretions are inhibited ([20, 21]). Thus, this model assumes that gastric juices are only added until the beginning of the emptying. The amount of gastric juices produced in response to a given meal is still not entirely known, but for this model, it is taken to be twice the volume of the ingested meal, as reported in [22]. Once emptying begins, there is no more dilution with gastric juices, so the process is seen, at both times, as a semi-batch process. The time needed to charge the stomach is considered negligible, as it is too short (15–20 min) compared to the total digestive time (approximately 3 h). Also, since the output of the stomach is too small during the *lag* phase, it is assumed to be negligible and, therefore, the pylorus is considered an on-off valve. The process is deemed to occur at constant temperature due to the thermal regulation of the human body.

**Fig 1:**
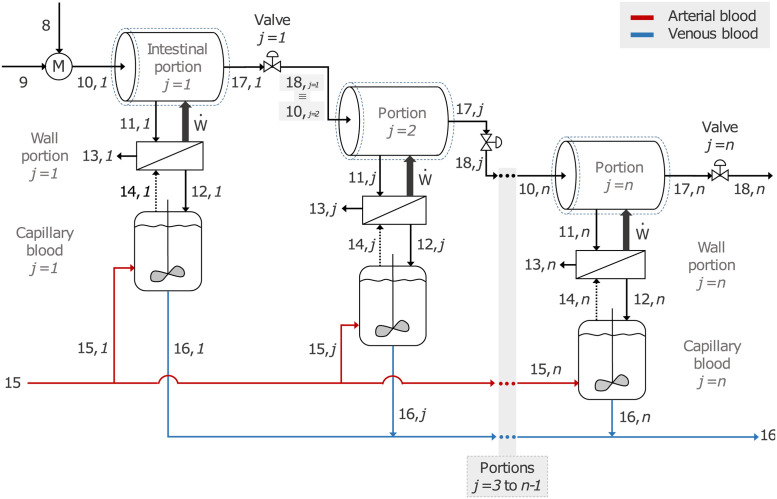
Modeling hypothesis for the small intestine representing a series of stirred tank reactors with elastic walls and connected by valves.

The small intestine is modeled as a set of stirred tanks, each with an elastic, semipermeable wall [23], connected to the adjacent tank through a valve that regulates the outflow according to the pressure drop between the two tanks. A detailed diagram for the modeling hypothesis of the small intestine is shown in Fig 1. Considering the elasticity of the wall of the tanks, a relationship between deformation and the normal force applied against the wall is established. Hence, such force determines the pressure of the fluid contained in the tank. Even though the cross-sectional area would initially be calculated using the diameter of the intestinal lumen, the pressure increase is related to muscle contractions, which, in contrast, diminishes the cross-sectional area. This effect is considered in the function that describes the opening of the valves. It is assumed that 10% of the absorbed glucose is invested in the peristaltic movements of the small intestine [7].

Additional considerations of the modeling hypothesis are as follows: i) all the polysaccharides, proteins and lipids are taken as a standard molecule, formed by 1000 units of glucose, 154 amino acids, and 3 esterified oleic acid units, respectively; ii) Even though water and some drugs can be absorbed in the stomach, nutrient absorption is negligible. Therefore, absorption is considered to happen exclusively in the small intestine.

#### Process system definition

**Fig 2:**
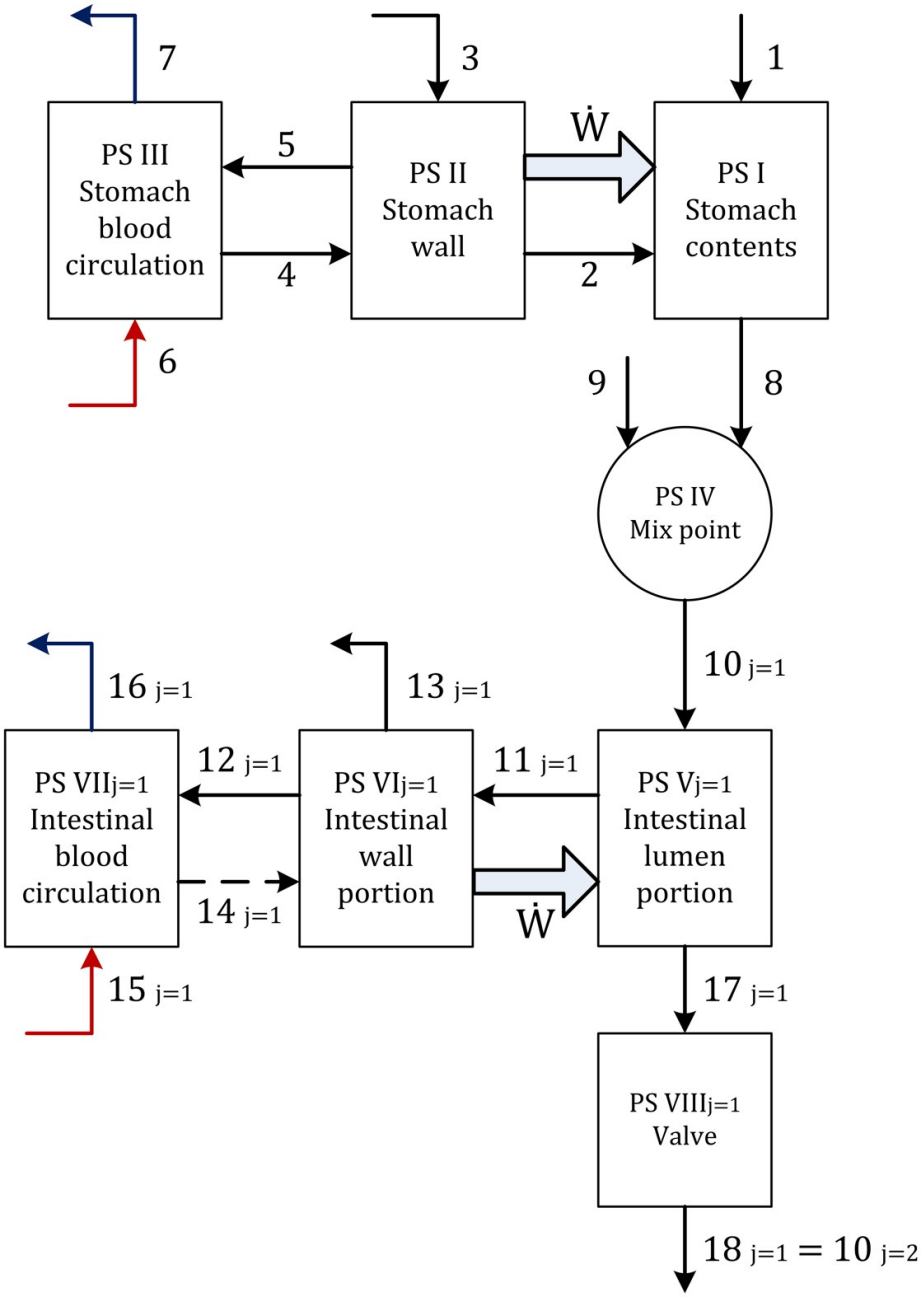
Block diagram of the model. The components of each stream are detailed in Section 2.

The process systems (PS) are defined according to the exchange of the substances of interest. Eight PS are defined for the current process as explained below, and they are all illustrated in Fig 2.

*PS*_*I*_ corresponds to the gastric mass contained in the stomach. Ingested food coming from the esophagus is represented by stream 1, gastric juices correspond to stream 2, and the chyme coming out of the stomach is stream 8.

*PS*_*II*_ corresponds to the stomach wall. Stream 3 carries the reactants needed to produce gastric juices. Stream 4 represents the reactants required to carry out glucose oxidation in the stomach wall, while the sub-products of that reaction (carbon dioxide and water) are delivered to the venous bloodstream in stream 5. The energy resulting from glucose oxidation is transferred to the *PS*_*I*_ (thick arrow). The blood that supplies the stomach wall is *PS*_*III*_, which receives arterial blood from the arterial celiac trunk through stream 6 and drains venous blood through stream 7.

*PS*_*IV*_ corresponds to a hypothetical mixing point where chyme emptied from the stomach is perfectly mixed with the pancreatic juices and bile entering through stream 9. Stream 10 represents the homogeneous mixture entering the small intestine.

*PS*_*V*_ represents the content of an intestinal lumen partition. Stream 11 represents the nutrients absorbed through the intestinal wall, and stream 17 represents the chyme flow passing from the current intestinal partition *j* to the next one *j+1*. The system’s energy input is given by peristalsis (thick arrow).

*PS*_*VI*_ represents a partition of the intestinal wall. A portion of the absorbed glucose is assumed to be used by this system to carry out peristaltic contractions, expressed as an energy expenditure with a thick arrow. Stream 13 represents a portion of the absorbed water, along with fatty acids and glycerol. In contrast, absorbed glucose, amino acids, electrolytes and remaining water pass to capillary blood through stream 12. If there is no absorption, the intestinal wall uses glucose from the capillary blood, represented by stream 14 as a dashed line.

*PS*_*VII*_ represents the intestinal blood irrigation. Here, arterial blood represented by stream 15 is mixed with glucose, amino acids, water, and electrolytes absorbed in stream 12. Stream 16 represents the venous capillary blood.

*PS*_*VIII*_ corresponds to the valve connecting the tank that represents the *j* intestinal partition to the *j+1* tank. Stream 18 represents the chyme outflow of the first tank, which corresponds to the inflow to the second one.

### Model construction

#### Application of the conservation principle

Mass balances are applied over the process systems to get the basic structure of the PBSM. These balances are presented in molar units for the systems in which chemical reactions take place, and in mass units otherwise. *PS*_*II*_ is the exception as balances are presented in mass units, as a consumption expression giving the reaction rate for glucose combustion instead of a kinetic expression. Not all of the mass and energy balances are presented here, only those that provide non redundant information regarding the process. Abbreviations of the substances of interest considered in the different process systems are detailed in Table 1.

**Table 1:** Substances of interest considered in the gastrointestinal (GI) model, symbolized as *s*.

Substance *s*	Definition	Substance *s*	Definition
CHO	Carbohydrates	CHO*	Shortened carbohydrates
G	Glucose	Pro*	Shortened proteins
Lip	Lipids	PA	Pancreatic amylase
FA	Fatty acids	PP	Pancreatic protease
Pro	Proteins	PL	Pancreatic lipase
AA	Amino acids	PA − In	Inactive pancreatic amylase
GL	Gastric lipase	PL − In	Inactive pancreatic lipase
GP	Gastric protease	PP − In	Inactive pancreatic protease
Gly	Glycerol	GJ	Gastric juices
F	Fiber	W	Water

- *PS*_*I*_—Stomach content: As explained earlier, this process system is always seen as a semi-batch process with no absorption taking place through the stomach walls.

**Total mass balance**.

$$\begin{array}{c} \frac{dN_{I}}{dt}={\dot{n}}_{2}-{\dot{n}}_{8}+\sum_{s}(\sigma_{s,I}\cdot r_{s,I}) \end{array}$$ (1)

where *N*_*I*_ represents the moles in the process system I, ${\dot{n}}_{2}$ indicates the molar flow entering the stomach through the stomach walls, ${\dot{n}}_{8}$ is the molar flow leaving the stomach due to gastric emptying. Moreover, *σ*_*s*,*I*_ and *r*_*s*,*I*_ indicate the stoichiometric coefficient and the reaction rate for the substance *s*, respectively. The substances of interest *s* considered for this PS are: CHO, G, Lip, FA, Pro, AA, F, GL, GP, GJ, and W. Finally, Eq 1 represents the variation over time of the moles present in the stomach due to dilution, gastric emptying, and enzymatic reactions.

**Component mass balance**. The following expression is obtained, after applying a mass balance for every substance *s* in the stomach

$$\begin{array}{c} \frac{dN_{s_{I}}}{dt}=x_{s_{2}}\;{\dot{n}}_{2}-x_{s_{8}}\;{\dot{n}}_{8}+\sigma_{s,I}\;r_{s,I} \end{array}$$ (2)

where $N_{s_{I}}$ represents the moles of *s* present in the *PS*_*I*_, and $x_{s_{2}}$ is the molar fraction of each substance in the stream containing gastric juices (it has a value of 1 for gastric juices and 0 for all of the other substances). Finally, $x_{s_{8}}$ is the molar fraction of each substance in the stomach outflow, which under the assumption of perfect mixing, is equal to the molar fraction inside the stomach at every instant.

**Total energy balance**. An energy balance is developed for a stationary state between the departure and arrival points of the gastric mass through the assumed piping system [11]. The continuity of the mass flow in the pipe circuit means that pressures, densities, flow regime factors, velocities, and heights do not change between the two considered points. Therefore, the energy balance can be reduced to the following expression:

$$\begin{array}{c} \dot{W}=\frac{1}{\eta }({\dot{m}}_{I}\cdot h_{f}) \end{array}$$ (3)

where $\dot{W}$ represents the energetic supply from the stomach walls, *η* is the efficiency to perform the mechanical work, ${\dot{m}}_{I}$ is the mass flow inside the stomach, and *h*_*f*_ represents the friction losses that the mass flow experiences while moving from the departure point to the arrival point of the assumed pipe circuit. This term was determined by using a mathematical expression for each of the pipes from the circuit, and it is broadly described in [11].

- *PS*_*II*_—Stomach Wall: The process occurring in this PS is seen as continuous. The only reaction considered is glucose combustion, which represents the thermodynamic state change resulting from the glucose oxidation pathway occurring in live tissues.

**Total mass balance**. The total mass balance for the *PS*_*II*_ is developed assuming that the stomach wall neither loses nor gains mass over the process. Enzymatic secretions from the stomach wall are not considered and enzymes are assumed to already be in the stomach from the beginning of the process. Finally, the mass flow of stream 3, which carries the reagents necessary to produce gastric juices, is assumed to be equal to the mass flow of stream 2, which corresponds to gastric juices. The balance is therefore written as:

$$\begin{array}{c} {\dot{m}}_{5}={\dot{m}}_{4} \end{array}$$ (4)

**Component mass balance**. Even though this PS receives and processes multiple substances (glucose, oxygen, carbon dioxide, and water), the only mass balance for glucose that brings useful information to the model is the following

$$\begin{array}{c} {\dot{m}}_{4}=\frac{{\hat{r}}_{G_{II}}}{w_{G_{4}}} \end{array}$$ (5)

where ${\hat{r}}_{G_{II}}$ represents the reaction rate for glucose combustion in mass units, and $w_{G_{II}}$ is the mass fraction of glucose in the stream containing the required reactants.

**Total energy balance**. The mechanical work performed by the system can be determined using the balance of thermal energy while considering the mechanical effects $\dot{W}$. For this balance, it was assumed, as mentioned above, that the human body has a perfect control system for temperature regulation. Therefore, the total energy change in this system is considered to be 0. As mass flows ${\dot{m}}_{2}$ and ${\dot{m}}_{3}$ are considered equal in both magnitude and composition, their enthalpies are also equivalent. Finally, the mass flow for the streams ${\dot{m}}_{4}$ and ${\dot{m}}_{5}$ is equal, and, after evaluating the enthalpy of each stream, it was concluded that their energetic content is roughly equal. Under these assumptions, the energy balance can be written as:

$$\begin{array}{c} {\hat{r}}_{G_{II}}=\frac{1}{\Delta {\bar{H}}_{r_{G_{II}}}}\dot{W} \end{array}$$ (6)

where $\dot{W}$ represents the mechanical power of the work done by the stomach wall, and $\Delta {\bar{H}}_{r_{G_{II}}}$ represents the specific molar heat of reaction for glucose combustion.

- *PS*_*III*_—Stomach Blood Irrigation: No reactions occur in this PS, therefore, balances are developed in mass units.

**Total mass balance**. Considering no accumulation within this PS, and bearing in mind that mass flows ${\dot{m}}_{4}$ and ${\dot{m}}_{5}$ are equal, means that mass flows ${\dot{m}}_{6}$ and ${\dot{m}}_{7}$ are equal as well. Thus, the total mass balance is given by

$$\begin{array}{c} \frac{dM_{III}}{dt}=0\;\Rightarrow M_{III}=Constant \end{array}$$ (7)

where *M*_*III*_ is the total blood mass in the vessels irrigating the stomach, which, as evinced, has a constant value. This value was estimated based on the literature [24].

**Component mass balance**. As with *PS*_*II*_, the substance of greatest interest on this PS is also glucose. For the development of said balance, perfect mixing is assumed, which means that $M_{G_{III}}=M_{III}\frac{dw_{G_{7}}}{dt}$, rendering:

$$\begin{array}{c} \frac{dw_{G_{7}}}{dt}=\frac{1}{M_{III}}(w_{G_{6}}\;{\dot{m}}_{6}-w_{G_{4}}\;{\dot{m}}_{4}-w_{G_{7}}\;{\dot{m}}_{7}) \end{array}$$ (8)

where ${\dot{m}}_{k}$ represents the mass flow of the stream *k*, and $w_{G_{k}}$ represents the mass fraction of glucose in the same stream.

- *PS*_*IV*_—Mixing point: The mixing of ingested food with bile and pancreatic juices in *PS*_*IV*_ is analyzed as a continuous process. Since no chemical reactions are considered here, the mass balances can be expressed in mass units.

**Total mass balance**. The total mass balance over *PS*_*IV*_ gives information on the mass flow resulting from mixing the ingested food emptied from the stomach (${\dot{m}}_{8}$) with pancreatic juices and bile (${\dot{m}}_{9}$), a process taking place in the first portion of the duodenum. Since no mass is accumulated in the hypothetical mixing volume within the duodenum, then $\frac{dM_{IV}}{dt}=0$. The resulting expression, solving for the mixture mass flow entering the duodenum (${\dot{m}}_{10,1}$), is the following algebraic equation

$$\begin{array}{c} {\dot{m}}_{10,1}={\dot{m}}_{8}+{\dot{m}}_{9} \end{array}$$ (9)

**Component mass balance**. Given that pancreatic juices and bile dilute the ingested food and add new substances to it, it is important to determine the composition of the mass flow starting to pass into the small intestine to calculate the digestion and absorption rates in the following PSs. The mass fraction of each component ($w_{s_{10,1}}$) exiting the *PS*_*IV*_ is determined by

$$\begin{array}{c} w_{s_{10,1}}=\frac{w_{s_{8}}{\dot{m}}_{8}+w_{s_{9}}{\dot{m}}_{9}}{{\dot{m}}_{10,1}} \end{array}$$ (10)

where $w_{s_{8}}$ is the mass fraction of components of the ingested food and $w_{s_{9}}$ is the pancreatic juices and bile emptied into the duodenum.

- Balances for the *PS*_*V*_—Intestinal lumen portion: Gastric mass flow is relatively slow compared to the length of the small intestine. Therefore, each portion *j* is assumed to be short enough to consider uniform properties throughout the contained gastric mass. Building the model makes it possible to account for the axial change of properties such as concentration, density, and pressure over time, all influenced by digestion, absorption, and mass flow velocity rates.

Thus, the analysis of the portions or instances *j* of this *PS* is performed by considering them as individual continuously-stirred tank reactors with two distinctive characteristics: *i)* the walls of the tanks are elastic, and thus their volumes are variable, and *ii)* there can be either positive or negative accumulation of gastric mass.

**Total mass balance**. The balance is expressed in terms of the change of the total mass contained in an elastic tank *M*_*V*,*j*_ as

$$\begin{array}{c} \frac{dM_{V,j}}{dt}={\dot{m}}_{10,j}-{\dot{m}}_{11,j}-{\dot{m}}_{17,j} \end{array}$$ (11)

where ${\dot{m}}_{10,j}$ is the mass flow of gastric mass entering a tank *j*, ${\dot{m}}_{11,j}$ the rate of mass absorption in each portion *j* towards the portal system, and ${\dot{m}}_{17,j}$ the mass flow of chyme moving on to the next portion of the intestine. In turn, a total volume balance is applied to each tank, assuming that only radial deformation is significant for the intestinal wall. Consequently, the length of each cylindrical tank remains constant. While this may not be true locally for a single portion, contraction and relaxation of muscles along the small intestine cause simultaneous shortening and elongation, altogether assumed to cancel each other out [25]. Applying the chain rule over the total mass balance gives

$$\begin{array}{c} \frac{dM_{V,j}}{dt}=\frac{d(\rho_{V,j}V_{V,j})}{dt}=V_{V,j}\frac{d\rho_{V,j}}{dt}+\rho_{V,j}\frac{dV_{V,j}}{dt} \end{array}$$

where *ρ*_*V*,*j*_ is the density of chyme and *V*_*V*,*j*_ is the volume of a tank. Subsequently, after replacing in Eq 11, the change in the volume of a tank is expressed as follows

$$\begin{array}{c} \frac{dV_{V,j}}{dt}=\frac{1}{\rho_{V,j}}({\dot{m}}_{10,j}-{\dot{m}}_{11,j}-{\dot{m}}_{17,j}-V_{V,j}{\dot{\rho }}_{V,j}) \end{array}$$ (12)

which successively gives place to an expression for the change of the radius of a transverse section of *PS*_*V*_: *r*_*j*_, shown in Eq 13. Assuming each portion of *PS*_*V*_ as a cylinder, the differential change can be written as

$$\begin{array}{c} \frac{dV_{V,j}}{dt}=\frac{d(\pi r_{j}^{2}L_{V})}{dt}=2\pi L_{V}r_{j}\frac{dr_{j}}{dt} \end{array}$$

where *L*_*V*_ is the length of an elastic tank. By replacing $\frac{d(\pi r_{j}^{2}L_{V})}{dt}$ in the total volume balance, the resulting expression for a change in the radius of a cylindrical intestinal portion is

$$\begin{array}{c} \frac{dr_{j}}{dt}=\frac{1}{2\pi L_{V}r_{j}\rho_{V,j}}({\dot{m}}_{10,j}-{\dot{m}}_{11,j}-{\dot{m}}_{17,j}-V_{V,j}{\dot{\rho }}_{V,j}) \end{array}$$ (13)

This expression, in particular, allows flow and hydrodynamic pressure calculations, further detailed for *PS*_*V*_ and *PS*_*VIII*_ in the section where constitutive and assessment equations are presented.

Lastly, since chyme composition changes during gastric emptying digestion and absorption processes, the density of the multi-component, homogeneous chyme is determined by clearing $\frac{d\rho_{V,j}}{dt}=\frac{1}{V_{V,j}}(\frac{dM_{V,j}}{dt}-\rho_{V,j}\frac{dV_{V,j}}{dt})$ from the previously defined chain rule, which after being replaced in Eq 11 gives

$$\begin{array}{c} \frac{d\rho_{V,j}}{dt}=\frac{1}{V_{V,j}}({\dot{m}}_{10,j}-{\dot{m}}_{11,j}-{\dot{m}}_{17,j}-\rho_{V,j}{\dot{V}}_{V,j}) \end{array}$$ (14)

Note that on the right-side of Eqs 12 and 14, the terms $\frac{dV_{V,j}}{dt}$ and $\frac{d\rho_{V,j}}{dt}$ were re-written as ${\dot{V}}_{V,j}$ and ${\dot{\rho }}_{V,j}$, respectively, as they are parameters for the model.

**Component mass balance**. In this *PS*, the component mass balances and their derived molar concentration and mass fraction balances are described for the substances *s*: AA, G, FA, W, Gly, CHO, Pro, Lip, CHO*, Pro*, PA, PP, PL, PA-In, PP-In, PL-In. Firstly, a general *s* component mass balance over *PS*_*V*_, including the mass flow of input and output streams, chemical reactions, and absorption terms, is expressed as

$$\begin{array}{c} \frac{dM_{s_{V,j}}}{dt}=w_{s_{10,j}}{\dot{m}}_{10,j}-{\dot{n}}_{s_{11,j}}M_{s}-w_{s_{17,j}}{\dot{m}}_{17,j}+V_{V,j}M_{s}\sum_{i}r_{s_{i,j}} \end{array}$$ (15)

where $M_{s_{V,j}}$ is the mass of the component *s* in a portion *j* of the *PS*_*V*_, $w_{s_{10,j}}$ the mass fraction of *s* in the stream 10 entering the *j*–*th* tank, ${\dot{n}}_{s_{11,j}}$ is the term for the molar rate of intestinal absorption of *s* in the *j*–*th* tank, $M_{s}$ is the molar mass of *s*, $w_{s_{17,j}}$ is the mass fraction of *s* in the stream 17 exiting the *j*–*th* tank, and $r_{s_{i,j}}$ is the molar rate of reaction of the component *s* in the reaction *i* = 1…6, which refer to the digestion and enzyme deactivation reactions, taking place in the chyme within the tank *j*. Chemical reaction and absorption rate terms for each component are further explained in the section where constitutive and assessment equations are presented.

The general balance of mass components is then expressed in terms of molar concentration ($C_{s_{V,j}}$) and mass fraction ($w_{s_{V,j}}$), to allow the calculation of mass flows, reaction, and absorption rates. To express it in terms of the molar concentration, $M_{s_{V,j}}$ is replaced by $M_{s}C_{s_{V,j}}V_{V,j}$ in the differential. By applying the chain rule and solving the differential for the molar concentration of *s* and writing $\frac{dV_{V,j}}{dt}$ as ${\dot{V}}_{V,j}$

$$\begin{array}{c} \frac{dC_{s_{V,j}}}{dt}=\frac{1}{M_{s}V_{V,j}}(\frac{dM_{s_{V,j}}}{dt}-M_{s}C_{s_{V,j}}{\dot{V}}_{V,j}) \end{array}$$

Finally, by replacing Eq 15 in the latter, the general expression of the change of molar concentration of *s* is obtained as

$$\begin{array}{c} \frac{dC_{s_{V,j}}}{dt}=\frac{1}{M_{s}V_{V,j}}\times \\ \left(w_{s_{10,j}}{\dot{m}}_{10,j}-{\dot{n}}_{s_{11,j}}M_{s}-w_{s_{17,j}}{\dot{m}}_{17,j}+V_{V,j}M_{s}\sum_{i}r_{s_{i,j}}-M_{s}C_{s_{V,j}}{\dot{V}}_{V,j}\right) \end{array}$$ (16)

Similarly, to express the general balance of mass components in terms of mass fraction, it suffices to define $M_{s_{V,j}}=M_{V,j}w_{s_{V,j}}$ and apply the chain rule, the differential is solved for the mass fraction of *s*. Replacing $\frac{dM_{V,j}}{dt}={\dot{M}}_{V,j}$ yields

$$\begin{array}{c} \frac{dw_{s_{V,j}}}{dt}=\frac{1}{M_{V,j}}(\frac{dM_{s_{V,j}}}{dt}-w_{s_{V,j}}{\dot{M}}_{V,j}) \end{array}$$

Lastly, if Eq 15 is replaced for the $\frac{dM_{s_{V,j}}}{dt}$ term in the last expression, the change in the mass fraction is defined as

$$\begin{array}{c} \frac{dw_{s_{V,j}}}{dt}=\frac{1}{M_{V,j}}\times \\ \left(w_{s_{10,j}}{\dot{m}}_{10,j}-{\dot{n}}_{s_{11,j}}M_{s}-w_{s_{17,j}}{\dot{m}}_{17,j}+V_{V,j}M_{s}\sum_{i}r_{s_{i,j}}-w_{s_{V,j}}{\dot{M}}_{V,j}\right) \end{array}$$ (17)

- *PS*_*VI*_—Intestinal wall portion: This *PS* is analyzed as a continuous filtration process, in which several substances of the chyme that flow along the intestinal lumen (*PS*_*V*,*j*_) are selectively transferred through a semipermeable membrane to cells of the intestinal wall and to the interstitium.

**Total mass balance**. Since the cells that are part of the intestinal wall consume nutrients to carry out their metabolic functions, mainly during the postprandial state [26], two additional terms were included in the input and output mass flows of this *PS* in the following total mass balance.

$$\begin{array}{c} \frac{dM_{VI,j}}{dt}={\dot{m}}_{11,j}+{\dot{m}}_{14,j}-{\dot{m}}_{12,j}-{\dot{m}}_{13,j}-r_{s_{VI,j}} \end{array}$$

where ${\dot{m}}_{12,j}$ is the mass flow of components that pass into the bloodstream (assumed to be glucose, amino acids, and water), ${\dot{m}}_{13,j}$ is the mass flow of components drained by the lymph (assumed to be glycerol and fatty acids), ${\dot{m}}_{14,j}$ the mass flow of glucose for cell uptake, and $r_{s_{VI,j}}$ the rate of consumption of *s* by each portion *j* of the small intestine wall. Even though the energy expenditure of the small intestine implies the consumption of different nutrients, the primary purpose of the model is to determine the rate of glucose absorption. Therefore, only glucose consumption is considered in this *PS* and $r_{G_{VI,j}}\neq 0$. With no mass accumulation occurring within this PS, the equation can be rewritten as presented in Eq 18, which is further solved for ${\dot{m}}_{12,j}$, the stream that carries the substances (AA, G and W) absorbed into the bloodstream.

$$\begin{array}{c} {\dot{m}}_{12,j}={\dot{m}}_{11,j}+{\dot{m}}_{14,j}-{\dot{m}}_{13,j}-r_{s_{VI,j}} \end{array}$$ (18)

**Component mass balance**. Similarly, the component mass balance is now performed for the absorbed components (glucose, amino acids, fatty acids, water, and glycerol), considering there is no mass accumulation,

$$\begin{array}{c} {\dot{m}}_{s_{12,j}}={\dot{n}}_{s_{11,j}}M_{s}+w_{s_{14,j}}{\dot{m}}_{14,j}-w_{s_{13,j}}{\dot{m}}_{13,j}-r_{s_{VI,j}} \end{array}$$ (19)

where ${\dot{m}}_{s_{12,j}}=w_{s_{12,j}}{\dot{m}}_{12,j}$ is the mass flow of the component *s* in the stream *k* = 12, and $w_{s_{k,j}}$ are the mass fractions of *s* in the streams *k* = 13, 14. It is important to note that for *s* ≠ *G*, the mass fractions $w_{s_{14,j}}=0$ and $w_{G_{14,j}}=1$, as cell uptake of nutrients in this *PS* was only considered for glucose.

- *PS*_*VII*_—Intestinal blood circulation: In this *PS*, a continuous mixing process in the blood that supplies the intestine is considered.

**Total mass balance**. No mass is accumulated inside the mixing tanks and no chemical reactions are taken into consideration, therefore $\frac{dM_{VII,j}}{dt}=0$. Since mass flows ${\dot{m}}_{12,j}$, ${\dot{m}}_{14,j}$ and ${\dot{m}}_{15,j}$ are known, the total mass balance in *PS*_*VII*_ is expressed in terms of the mass flow of blood draining the small intestine

$$\begin{array}{c} {\dot{m}}_{16,j}={\dot{m}}_{12,j}+{\dot{m}}_{15,j}-{\dot{m}}_{14,j} \end{array}$$ (20)

Moreover, the sum of all the ${\dot{m}}_{16,j}$ mass flows along the small intestine represents the mass flow of the superior mesenteric vein ${\dot{m}}_{16}$, which in turn transports glucose into the portal vein.

$$\begin{array}{c} {\dot{m}}_{16}=\sum_{j}{\dot{m}}_{16,j} \end{array}$$ (21)

**Component mass balance**. The component mass balance for glucose is of particular interest as it is directly linked to answering the question posed for the model. Given that no mass is accumulated, $\frac{dM_{G_{VII,j}}}{dt}=0$, the balance gives rise to an algebraic equation. The dynamic change of glucose mass in the stream 16, *j* is then defined as

$$\begin{array}{c} w_{G_{16,j}}{\dot{m}}_{16,j}={\dot{m}}_{G_{16,j}}=w_{G_{12,j}}{\dot{m}}_{12,j}+w_{G_{15,j}}{\dot{m}}_{15,j}-w_{G_{14,j}}{\dot{m}}_{14,j} \end{array}$$ (22)

As mentioned above, $w_{G_{14,j}}=1$, because only glucose is being considered for cell nutrient uptake in the mathematical model. Also, the product $w_{G_{16,j}}{\dot{m}}_{16,j}$ is the mass flow of glucose in the stream 16, which can be simply written as ${\dot{m}}_{G_{16,j}}$, and the mass fraction $w_{G_{15,j}}$ derives from the systemic glucose concentration, as explained in the section where constitutive and assessment equations are presented.

Before determining glucose concentration in the stream 16, the individual mass flows of glucose from each portion *j* must be added up

$$\begin{array}{c} {\dot{m}}_{G_{16}}=\sum_{j}{\dot{m}}_{G_{16,j}} \end{array}$$ (23)

Finally, the mass fraction of glucose in stream 16 is expressed as

$$\begin{array}{c} w_{G_{16}}=\frac{{\dot{m}}_{G_{16}}}{{\dot{m}}_{16}} \end{array}$$ (24)

Given the widespread acceptance of *mg*/*dL* concentration units to measure glucose levels in the blood, the last expression is posed as

$$\begin{array}{c} C_{G_{16}}=\frac{1}{10}\;\rho_{16}\;w_{G_{16}}\;\left[=\right]\;mg/dL \end{array}$$ (25)

Moreover, the current model can include expressions for other components such as amino acids, glycerol, and water absorbed in the bloodstream. This task would imply the definition of more specific rates of amino acid absorption, and a particular partition coefficient between blood and lymph for water, glycerol, and low-weight fatty acids.

- *PS*_*VIII*_—Valve: The muscles in the intestinal wall regulate mass flow between portions of the small intestine. One way to account for the regulation of chyme flow, as well as other factors that slow down the flow of chyme, such as friction, is to model the flow through a static valve as a continuous process.

**Total mass balance**. Since this *PS* represents a flow area, there is no mass accumulation, rendering $\frac{dM_{VIII,j}}{dt}=0$, thus ${\dot{m}}_{17,j}={\dot{m}}_{18,j}$. However, stream ${\dot{m}}_{18,j}$ is only considered for the last portion *j*, then, the stream exiting the valve of slice *j* immediately enters the next portion of small intestine *j* + 1. Thus, ${\dot{m}}_{18,j}={\dot{m}}_{10,j+1}$ and

$$\begin{array}{c} {\dot{m}}_{17,j}={\dot{m}}_{10,j+1} \end{array}$$ (26)

**Component mass balance**. Similarly, compositions remain unchanged while the stream flows across the flow area, rendering

$$\begin{array}{c} w_{s_{17,j}}=w_{s_{10,j+1}} \end{array}$$ (27)

**Total energy balance**. Repeated contraction and relaxation of the smooth muscle tissue in the small intestine propagates caudally as a peristaltic wave, which facilitates chyme digestion and absorption by generating flow patterns and exerting stress that aids breakdown, mixing, and transport [25]. This movement favors churning and mixing food particles in chyme rather than its transport to maximize nutrient absorption throughout the segments of the small intestine. For this reason, the chyme flow velocity is low, and the flow regime is primarily laminar, although contractions lead to fluid circulation and the production of vortices.

For this *PS*, peristaltic muscle contractions are assumed to produce both a pressure difference and a variation in flow area, resembling a fluid flow through a static valve. Thus, an energy balance would provide insights into the calculation of the chyme flow from one portion of the small intestine to another, due to peristalsis. For this purpose, thermal effects were neglected, given that body temperature is regulated within a narrow interval. However, a mechanical energy balance provides information regarding friction effects and hydrodynamic pressure on chyme flow. Since no mass is accumulated in the valve, a mechanical energy balance for steady flow using Bernoulli’s principle is applied, setting departure and arrival points right before and after the valve, respectively. The area of valve connections is equal for both ends, therefore *v*_17,*j*_ = *v*_10,*j*+1_. Assuming a uniform and steady flow, negligible height changes, and replacing the definition of flow coefficient (*c*_*v*_, further explained in [27]), considering $\dot{V}=A_{flow}\;v$, the mechanical energy balance (MEB) renders

$$\begin{array}{c} {\dot{m}}_{10,j+1}=c_{v_{j}}\rho_{V,j}\sqrt{P_{V,j}-P_{V,j+1}} \end{array}$$ (28)

#### Basic structure of the model

The basic structure of the model is obtained by choosing those equations that bring up the most relevant, non-redundant information. For the current model, these are: (1), (2) and (3), taking into account that Eq (2) generates 11 equations, one for every substance *s*. Also, Eqs (5), (6), (7), (8), (9) and (10), where the latter generates 17 equations, one for every component *s* entering the first portion *j* of the intestinal lumen. Eqs (11), (12), (13), (14), (15), (16) and (17), where the last three equations generate 20 equations each, one for every component *s* considered for Eq (10), but adding inactive components PA-In, PP-In, PL-In. In addition, Eq (19), which generates 5 equations, one for each component *s* absorbed into the portal system. Likewise, Eqs (20), (21), (22), (23), (24) and (25), all of them representing the appearance of glucose in the portal system, and Eqs (26), (27) and (28), where the second expression generates 20 more, one for every component *s* that is not digested or absorbed, and passes to the colon. The whole set of equations are presented in the S1 Appendix.

#### Determining variables, structural parameters, and structural constants

In this step, the model’s variables, structural parameters, and structural constants are established and described in Table 2.

**Table 2:** Variables, structural parameters and constants for each PS.

	Variables	Structural parameters	Constants
*PS*_*I*_	*N*_*I*_, $N_{s_{I}}$, $\dot{W}$	${\dot{n}}_{2}$ , ${\dot{n}}_{8}$, $x_{s_{8}}$, *r*_*s*,*I*_, ${\dot{m}}_{I}$, *h*_*f*_	$x_{s_{2}}$ , *σ*_*s*,*I*_, *η*
*PS*_*II*_	${\dot{m}}_{4}$ , ${\hat{r}}_{G_{II}}$	-	$w_{G_{4}}$ , $\Delta {\bar{H}}_{r_{G_{II}}}$
*PS*_*III*_	$w_{G_{7}}$	${\dot{m}}_{6}$ , ${\dot{m}}_{7}$, $w_{G_{6}}$	*M*_*III*_
*PS*_*IV*_	${\dot{m}}_{10,1}$ , $w_{s_{10,1}}$	${\dot{m}}_{8}$ , ${\dot{m}}_{9}$, $w_{s_{8}}$, $w_{s_{9}}$	-
*PS*_*V*_	*M*_*V*,*j*_, *V*_*V*,*j*_, *r*_*j*_, *ρ*_*V*,*j*_, $M_{s_{V,j}}$, $C_{s_{V,j}}$, $w_{s_{V,j}}$	${\dot{m}}_{11,j}$ , *L*_*V*_, ${\dot{n}}_{s_{11,j}}$, $w_{s_{14,j}}$, $r_{s_{i,j}}$	$M_{s}$
*PS*_*VI*_	*M*_*VI*,*j*_, $M_{s_{VI,j}}$	${\dot{m}}_{12,j}$ , ${\dot{m}}_{13,j}$, ${\dot{m}}_{14,j}$, $r_{s_{VI,j}}$, $w_{s_{12,j}}$, $w_{s_{13,j}}$	-
*PS*_*VII*_	${\dot{m}}_{16,j}$ , ${\dot{m}}_{16}$, ${\dot{m}}_{G_{16,j}}$, ${\dot{m}}_{G_{16}}$, $w_{G_{16}}$	${\dot{m}}_{15,j}$ , $w_{G_{12,j}}$, $w_{G_{14,j}}$, $w_{G_{15,j}}$, $w_{G_{15,j}}$	-
*PS*_*VIII*_	${\dot{m}}_{17,j}$ , $w_{s_{17,j}}$, ${\dot{m}}_{10,j+1}$	$w_{s_{10,j+1}}$ , $c_{v_{j}}$, *P*_*V*,*j*_, *P*_*V*,*j*+ 1_	-
**Total**	158	171	55

The index *s* indicates: G, CHO, Lip, FA, Pro, AA, W, F, GJ, GL, GP, PA, PP, PL, Gly, CHO*, Pro*, PA-In, PP-In, PL-In. The index *i* indicates the digestion reactions in the intestinal lumen: 1 = Carbohydrates digestion, 2 = Amylase pancreatic inactivation, 3 = Proteins digestion, 4 = Protease pancreatic inactivation, 5 = Lipids digestion, 6 = Lipase pancreatic inactivation.

#### Constitutive and assessment equations for structural and functional parameters

Generally, constitutive and assessment equations are algebraic equations of diverse origins, formulated and added to the conservation balances to include specific knowledge such as mass transfer and reaction rates, leading to a model with a deeper level of detail [28]. Every structural parameter may be defined with a constitutive or assessment equation. In turn, each parameter derived from these expressions is called a functional parameter and may be further defined by another constitutive equation, by a constant value from data from literature, or may be identified from real data.

The parametric identification procedure comprised testing parameter values to reduce model prediction error in contrast to experimental data. The resulting extended structure of the model arises from the underlying phenomena, available knowledge of the process, and the skill of the modeler. The selection of the most relevant constitutive and assessment equations is discussed below. Constitutive and assessment equations for defining the model’s structural parameters are reported in Table 5 in S1 Appendix. Constitutive equations for defining functional parameters are provided in Table 6 in S1 Appendix, while Table 7 in S1 Appendix introduces assessment equations and the model’s constants. These three tables are presented in the S1 Appendix. Notations A and I used in the tables refer to if the parameters were *assumed* and their value was fixed under different conditions for the entire simulation, or *identified*, respectively. *From the model* (M) means that the parameter is calculated by an equation that represents the dynamics of the system itself.

As mentioned in the modeling hypothesis, a set of stirred tanks with elastic walls was used as an analogy to represent the movement of chyme through the intestinal lumen. A link between the deformation of the small intestine wall and the hydrodynamic pressure (*P*_*V*,*j*_) of the fluid flowing from tank to tank can be established by relating a constitutive model of the strain energy density with the equilibrium of forces along the wall. The small intestine wall is well characterized by the Fung hyperelastic model [29, 30]. The hydrodynamic pressure can be calculated, in cylindrical coordinates, as stated in Eq 29 (further detailed in [31]).

$$\begin{array}{c} P_{V,j}=\;\frac{1}{4}C_{j}\;\text{exp}\;(a_{2_{j}}E_{zz}^{2})\times [\text{erfi}(\frac{A_{V,j}-A_{0_{V,j}}}{2A_{0_{V,j}}}\sqrt{a_{1_{j}}})-\text{erfi}(\frac{A_{V,j}-A_{0_{V,j}}}{2(A_{0_{V,j}}+A_{wall_{V,j}})}\sqrt{a_{1_{j}}})]\sqrt{\pi a_{1_{j}}} \end{array}$$ (29)

The small intestine has three different structural parts. The geometric and mechanical properties of the intestinal wall (Fung material parameters *C*_*j*_, $a_{1_{j}}$, $a_{2_{j}}$, wall thickness $h_{0_{j}}$, and tank radius $r_{0_{j}}$) vary along the path of chyme, as do kinetic expressions of digestion and absorption. Therefore, these parameters were fitted using smooth polynomial functions, as a function of the position along the small intestine, to produce subtler changes. Equations for functional parameters *C*_*j*_, $a_{1_{j}}$, and $a_{2_{j}}$ in Table 6 in S1 Appendix of the Fung constitutive model directly account for the change of elasticity along the intestinal wall, and were fitted by using data from [29]. Eq 43 and Eq 44 for $r_{0_{j}}$ and $h_{0_{j}}$, respectively (see Table 6 in S1 Appendix), describe the functional relation between the *basal* or *deflated* radius and thickness of the intestinal wall with respect to the position along the small intestine, and were fitted using data from [32]. Based on these equations, the parameter *L*_*V*_ (in the definition of *L*_*j*_) is the fixed length of a tank or portion and depends on the total length of the small intestine *L* and the number of tanks *n* in which the intestine is partitioned. In this case, *n* = 52, *L* = 5.2, and the lengths of duodenum, jejunum, and ileum are 0.2, 2.3 and 2.7, respectively. These values were assumed based on the average lengths of the small intestine measured in live healthy humans, as reported in [33].

The effects of more complex rheological phenomena taking place in the small intestine due to the high viscosity of chyme, laminar flow regime, and circular muscle contractions, which cause an area reduction that pushes the chyme to the next portion of the small intestine, are considered implicitly with the abstraction of the definition of the structural parameter $c_{v_{j}}$. For this reason, the inherent valve characteristic is defined quite differently, as a function of the pressure drop of the chyme across the hypothetical flow area separating two portions, weighted using the equivalent mean contraction pressure ${\bar{P}}_{cont}$.

Furthermore, during the postprandial state, peristaltic waves occur every 25 seconds approximately, last an average of 3.1 seconds and exert a pressure of about 2666 [34, 35]. For reasons of simplification, the net mechanical work exerted by one peristaltic wave is divided over its period of time to have an equivalent peristaltic wave with lower pressure and a constant muscle contraction by using the Pulse-Width Modulation (PWM) approach. In this sense, *τ* = 3.1*s* is the pulse width (duration of the ON state in a cycle or peristaltic wave) and *T* = 25*s* the total period of the peristaltic wave. Overall, the width of the peristaltic wave is increased from *τ* to *T*, at the expense of decreasing its amplitude (the average contraction pressure), yielding an equivalent mean contraction pressure, ${\bar{P}}_{cont}$, which is calculated by using the pressure *P*_*cont*_(*t*) exerted by the small intestine wall on the chyme. It has a constant value of 2666 for 3.1 seconds and then falls to zero until a new peristaltic wave generates after 25 seconds. In this simplified case, it would be enough to use the duty cycle *DC* to weigh the mechanical work exerted by the small intestine walls over the period of occurrence of a peristaltic wave [36].

As previously described, complete digestion of macronutrients and inactivation of digestive enzymes occur in *PS*_*V*_, setting the pace at which several bio-available nutrients are released. For reasons of simplification, a general expression for reaction rates is further detailed to determine the concentration of substances of interest for glucose homeostasis, such as glucose and gluconeogenic precursors (e.g., glycerol, alanine, glutamine). Reaction rates are calculated at a temperature of *T* = 310.

**Reaction *i* = 1: Carbohydrate digestion**. In this model, carbohydrates (CHO) are assumed to be simple amylose chains with an average molar mass $M_{CHO}$ equal to 180000*kg*/*kmol*. The hydrolysis reaction (reaction 1 in Table 3) releases a glucose molecule from one end of the amylose chain per water molecule, shortening its length progressively (producing CHO*). For reasons of simplification, the molar mass of the amylose chain is considered constant. In turn, a mass balance calculates the remaining equivalent moles of a hypothetical complete amylose chain. The expression for the reaction rate is adapted to take the form of first-order reaction kinetics, depending solely on the concentration of the pancreatic amylase enzyme. The underlying assumption is that enzyme concentration is limiting and much lower than that of the substrates, so the reaction order is zero for CHO concentration.

**Table 3:** Reaction rates of macro-nutrient digestion and enzyme inactivation in the small intestine.

*i*	Reaction	Rate expressions	Ref.
1	*CHO* + *H*2*O* → CHO* + *G*	$\begin{array}{l} r_{G_{1,j}}=\sigma_{G,1}k_{0_{1,z}}C_{PA_{V,j}}e^{\frac{-Ea_{1}}{R\;T}}\\ r_{W_{1,j}}=\frac{\sigma_{W,1}}{\sigma_{G,1}}r_{G_{1,j}}\\ r_{CHO_{1,j}}=\frac{\sigma_{CHO,1}}{\sigma_{CHO^{*},1}}\frac{1}{M_{CHO}}\left(M_{G}r_{G_{1,j}}+M_{W}r_{W_{1,j}}\right) \end{array}$	[27], M
2	*PA* → *PA* − *In*	$\begin{array}{l} r_{PA_{2,j}}=\sigma_{PA,2}\left(k_{0_{2,z}}C_{PA_{V,j}}e^{\frac{-Ea_{2}}{R\;T}}+k_{deg_{2,z}}C_{PP_{V,j}}\right)\\ r_{PA-In_{2,j}}=\sigma_{PA-In,2}\;r_{PA_{2,j}} \end{array}$	[27], M
3	*Pro* + *H*2*O* → Pro* + *AA*	$\begin{array}{l} r_{AA_{3,j}}=\sigma_{AA,3}k_{0_{3,z}}C_{PP_{V,j}}e^{\frac{-Ea_{3}}{R\;T}}\\ r_{W_{3,j}}=\frac{\sigma_{W,3}}{\sigma_{AA,3}}r_{AA_{3,j}}\\ r_{Pro_{3,j}}=\frac{\sigma_{Pro,3}}{\sigma_{Pro^{*},3}}\frac{1}{M_{Pro}}\left(M_{AA}r_{AA_{3,j}}+M_{W}r_{W_{3,j}}\right) \end{array}$	[27], M
4	*PP* → *PP* − *In*	$\begin{array}{l} r_{PP_{4,j}}=\sigma_{PP,4}k_{0_{4,z}}C_{PP_{V,j}}e^{\frac{-Ea_{4}}{R\;T}}\\ r_{PP-In_{4,j}}=\sigma_{PP-In,4}\;r_{PP_{4,j}} \end{array}$	[27], M
5	*Lip* + 3*H*2*O* → 3*FA* + *Gly*	$\begin{array}{l} r_{FA_{5,j}}=\sigma_{FA,5}k_{0_{5,z}}C_{PL_{V,j}}e^{\frac{-Ea_{5}}{R\;T}}\\ r_{Lip_{5,j}}=\frac{\sigma_{Lip,5}}{\sigma_{FA,5}}r_{FA_{5,j}}\\ r_{W_{5,j}}=\frac{\sigma_{W,5}}{\sigma_{FA,5}}r_{FA_{5,j}}\\ r_{Gly_{5,j}}=\frac{\sigma_{Gly,5}}{\sigma_{FA,5}}r_{FA_{5,j}} \end{array}$	[27], M
6	*PL* → *PL* − *In*	$\begin{array}{l} r_{PL_{6,j}}=\sigma_{PL,6}\left(k_{0_{6,z}}C_{PL_{V,j}}e^{\frac{-Ea_{6}}{R\;T}}+k_{deg_{6,z}}C_{PP_{V,j}}\right)\\ r_{PL-In_{6,j}}=\sigma_{PL-In,6}\;r_{PL_{6,j}} \end{array}$	[27], M

Abbreviations. M: From the model.

**Reaction *i* = 2: Pancreatic amylase inactivation**. Digestive enzymes in chyme are gradually inactivated along the small intestine predominantly due to pH changes, interactions with many substances in the chyme, and the presence of proteases, which partly degrade peptide bonds and alter structural integrity, comprising catalytic activity. The higher resistance of the pancreatic amylase against proteases makes it more stable than lipases and proteases [37]. A simplified description of the inactivation of pancreatic amylase (PA) is presented in Eq 2 of Table 3.

**Reaction *i* = 3: Protein digestion**. Proteins are digested through a hydrolysis reaction, which is simplified by assuming that reaction with one molecule of water releases one *average* amino acids and produces a shortened protein (Pro*), described in Eq 3 of Table 3. All the ingested proteins are assumed to be myosin, the most abundant of proteins found in meat [38], with an average molar mass $M_{Pro}$ equal to 493000*kg*/*kmol* [39]. Since the protein chain is continuously shortened, a mass balance is proposed to obtain a rate expression in terms of an equivalent, intact myosin molecule.

**Reaction *i* = 4: Protease inactivation**. Many proteases released along the gastrointestinal tract digest proteins in the small intestine. Trypsin was set as the equivalent average pancreatic protease for this model. Rate expression for the inactivation reaction depends solely on protease concentration (see Eq 4, Table 3).

**Reaction *i* = 5: Lipid digestion**. For this reaction, all lipids are considered to be triglycerides, more specifically triolein, which produces three units of oleic acid (FA, for fatty acid) and one of glycerol (Gly) upon its hydrolysis, catalyzed by pancreatic lipases. This reaction is shown in Eq 5, Table 3, and the reaction rate is described with a first-order expression, depending on the concentration of pancreatic lipase, assuming enzyme concentration to be significantly much lower than that of the substrates.

**Reaction *i* = 6: Pancreatic lipase inactivation**. Inactivation of pancreatic lipase, presented in Eq 6 of Table 3, is caused both by proteases and the interaction of these enzymes with other substances in the chyme. For this reason, the expression for the reaction rate depends on the concentration of both PL and PP, as shown in Eq 6, Table 3.

Regarding the absorption of nutrients into the bloodstream, glucose, amino acids, fatty acids, and water all pass through the wall of the small intestine in different ways. Luminal glucose is mainly absorbed across the brush membrane border through the Na^+^-glucose cotransporter SGLT1 and the GLUT2 carrier protein. The latter mechanism of glucose absorption is known as facilitated diffusive transport and is increasingly prevalent at higher glucose concentrations. The hydrophobicity of the membranes prevents strict passive or diffusive transport of glucose and tight junctions between enterocytes, leaving molecules no space to passively diffuse into the interstitium [40]. While the exclusive protein-mediated and Na^+^-codependent glucose transport could be deemed as a saturable transport mechanism, the increasing presence of GLUT2 proteins in the brush border membrane with higher glucose concentrations bypasses saturation. For instance, this could be mathematically represented by adding a passive transport term to a Michaelis-Menten absorption kinetics expression [41, 42]. Prior data from glucose absorption experiments in the human jejunum has been successfully fitted to glucose uptake expressions with both active and passive components [43]. Accordingly, this type of glucose uptake expression was included as a constitutive equation of this model, as shown in Eq 22, Table 5 in S1 Appendix. It is now known that the *passive* absorption term in this constitutive equation accounts for the effect of increasing GLUT2 proteins at the brush membrane border with higher glucose levels. Thus, this term refers to a facilitated diffusive transport mechanism rather than a passive one. Since glucose absorption rates differ along the small intestine, parameters were identified separately for the duodenum, jejunum, and ileum.

Although many amino acid transporters are present in the brush border membrane, the real scenario involves the absorption of di- and tri-peptides into enterocytes through oligopeptide transporters, succeeded by their break down into amino acids by peptidases [44]. Since hydrolysis of oligopeptides is very rapid and geared toward a more straightforward mathematical expression, only the absorption of an average, equivalent amino acid is being considered. Moreover, bearing in mind that the absorption of amino acids and peptides mainly occurs via an active, saturable transport mechanism, it would be sufficient for the goal of the model to use a simple Michaelis-Menten kinetic expression, to which L-alanine and glycine absorption data in human jejunum has been successfully fitted [45], as shown in Table 5 in S1 Appendix. Once again, the values of functional parameters arising from the absorption expression were independently identified for the duodenum, jejunum, and ileum.

Lipids and bile acids form mixed micelles within the intestinal lumen, facilitating transport across the unstirred diffusion barrier at the lumen-membrane interface, ensuring a sufficient concentration gradient for lipid uptake. Unlike absorption of more polar substances, transport of lipids across the brush border membrane does not exclusively rely on transporter proteins. Transport of short-chain fatty acids across the membrane may occur via simple passive diffusion, while the uptake of medium-chain, large-chain fatty acids, and other lipids takes place by active and facilitated transport mechanisms [46]. Despite the diversity of transfer mechanisms, types and sizes of lipids, the model is intended to produce a lower level of detail regarding lipid absorption. Thus, a more straightforward mass transfer expression following Fick’s first law of diffusion is proposed for oleic acid, the only fatty acid assumed to make part of the ingested meal (esterified in the form of triolein). The expression, further detailed in Table 5 in S1 Appendix, includes an assumed equilibrium concentration $C_{FA_{VI}}^{*}$ to set the concentration gradient that drives the uptake of fatty acids, considering that the chemical complexity within the cell renders such value indeterminable over time.

Water is absorbed throughout the three structural parts of the small intestine. This physiological process is heavily influenced by the osmolality of chyme [47], blood volume, and gastric emptying rate and therefore depends on a wide range of mechanical and neurohormonal stimuli occurring before, during and after the ingestion of a meal [21]. The osmolality of chyme is not considered as a variable to reduce the complexity of the constitutive equation that integrates water absorption with the model. The simulated scenario implies that its value is such that it produces a positive net water absorption from the lumen into the systemic circulation. Thus, this model does not account for the effect of altered intestinal motility due to the composition of the ingested meal, drinks, or drugs, nor any neuromuscular disorders. On average, water absorption in the small intestine of healthy humans ranges from 9 to 15 *mL* ⋅ *cm*^−1^ ⋅ *h*^−1^, following ingestion of various carbohydrate solutions [48]. From these data, water absorption percentages in the small intestine [49], and assuming that: *i)* the flow of chyme and other secretions in the small intestine ranges between 0 and 20 ⋅−1 and *ii)* the duodenal mass flow is a significant mechanical stimulus that is linearly related to the water absorption rate, the water absorption coefficients of the duodenum, jejunum, and ileum were identified and are presented in Table 5 in S1 Appendix.

**Fig 3:**
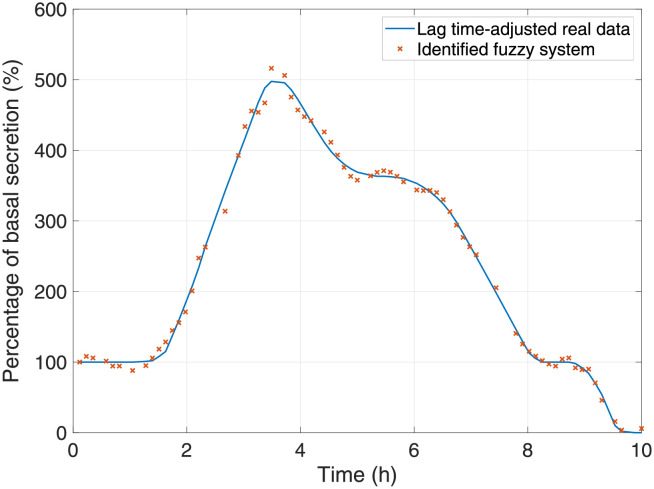
Percentage of basal secretion calculated by the identified fuzzy system. The data used for model identification were retrieved from [50].

The values of the total gastric mass emptied from the stomach and the mass fraction of each component are obtained by solving equations of the *PS*_*I*−*IV*_, described earlier and further detailed in [11]. This gastric mass flow is mixed in the duodenum with bile and pancreatic juices arriving through the hepatopancreatic duct to aid the digestion of partially digested chyme. Secretions of pancreatic enzymes and bile are estimated from a Takagi-Sugeno fuzzy model, identified based on data reported in [50], and obtained using the methodology described by [51]. This fuzzy model is set with thirteen rules and determines the percentage of secretions over the postprandial state with an interdigestive or basal secretion, which serves as the reference. For the meal previously detailed and its corresponding gastric emptying lag time, the percentages of basal secretion calculated based on the fuzzy model are shown in Fig 3.

Pancreatic and bile secretions are influenced by the concentration of nutrients in the duodenum and several neurohormonal signals stimulated by the sight and taste of food, the distension of both stomach and duodenum, and other factors such as stress or depression. Generally, these produce a pronounced peak in the rate of hepatopancreatic secretions, followed by a sustained, moderate secretion rate before transitioning into the interdigestive period, where secretions are minimum, or basal [50, 52]. In the fuzzy model, both the appearance of the peak over the postprandial period and the subsequent plateau can be slightly anticipated or delayed according to the lag time of gastric emptying.

The resulting percentage of basal secretion is then transformed into a mass flow by using a conversion factor, considering that, on average: *i)* interdigestive and maximum postprandial pancreatic juice flows are 0.25 and 4 *mL* ⋅ *min*^−1^, respectively [52], and *ii)* interdigestive and maximum postprandial flows of bile are 0.75 and 2.5 *mL* ⋅ *min*^−1^, sequentially [53, 54].

On the other hand, the concentration of pancreatic enzymes in the mixed bile and pancreatic juice is yet to be determined. The products of lipid digestion have been shown to be major stimulants of pancreatic enzyme secretions [55]. Thus, a relationship between the concentration of fatty acids and enzyme activity of pancreatic amylase was established. This relationship (Eq 16 in Table 6 in S1 Appendix) was formulated using a Michaelis-Menten-like expression, which assumes that cell receptors sensing fatty acids are saturable, with an added constant term representing the enzyme activity due to stimuli other than the presence of fatty acids in the duodenum. The expression was fitted using data on enzyme activity of secreted pancreatic amylase with various oleic acid concentrations [55], and it was converted into activities of equivalent pancreatic protease and lipase using amylase to lipase to trypsin ratios [50], all according to the current concentration of fatty acids in the first portion of the small intestine.

The cell glucose uptake rate which depends on the energetic expenditure of the small intestine wall was assumed as a 10% of absorbed glucose ${\dot{n}}_{G_{11,j}}$[7]. However, when no enteral glucose is present in the lumen, the intestinal wall sources glucose from the systemic circulation via ${\dot{m}}_{14,j}$, which was adjusted at a fixed consumption rate. Since arterial blood flow of the superior mesenteric artery is divided into smaller branches that locally irrigate all the portions of the small intestine, the mass fraction (and therefore, the molar concentration) of glucose remains unchanged, rendering $w_{G_{15}}=w_{G_{15,j}}$. This value is deemed the systemic glucose concentration, which will accordingly change in response to the glucose absorption rate into the blood of capillaries. Blood in the capillaries drains the small intestine, converging in the superior mesenteric vein, which delivers enteral glucose into the portal vein, with a glucose mass fraction equal to $w_{G_{16}}$. The glucose concentration data used to obtain $w_{G_{15}}$ were retrieved from [56] and, in turn, identified another fuzzy model (following the methodology described by [51]), which allows a sampling of the systemic glucose concentration at any given time.

The portal vein raises from gastric, splenic, superior mesenteric and inferior mesenteric veins, so that determining each mass flow and composition would considerably increase the model’s complexity. A more convenient and still realistic scenario is to assume the magnitude of the volume flow irrigating the small intestine equal to that of the portal vein, so the flow of the components being absorbed from the small intestine has a weighted impact on the composition due to the higher dilution that a larger volume flow produces. Thus, arterial flow ${\dot{m}}_{15}$ is calculated using the volume flow in the portal vein.

### Simulation of the computational model

#### Computational model construction

**Fig 4:**
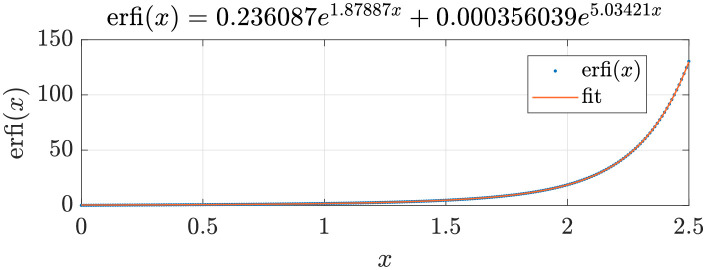
Fit of erfi(*x*) using an expression of the form *y* = *ae*^*bx*^+ *ce*^*dx*^. The graph presents the fitted function and evaluation of erfi(*x*).

Programming and simulation of the model were performed using MaTLab^®^. To significantly reduce computation time, the imaginary error function, erfi(x), was simplified by fitting within the interval *x* ∈ [0, 2], obtaining the expression erfi(*x*) = 0.236*e*^1.88*x*^ + 0.000356*e*^5.034*x*^, shown in Fig 4. The maximum absolute error and root-mean-square error are 0.21 and 0.0181, respectively. For *x* > 2, the former solver for erfi(*x*) in MaTLab^®^ is used.

The computational model is free (interested readers can obtain it from the corresponding author) and can be used to generate the simulations presented in the paper.

#### Comparison with data

The mathematical model responses were contrasted with data available in the literature. Experimental data from three studies [56–58] were used to compare the rate of glucose appearance in the portal system, and the inactivation percentage of pancreatic enzymes was fitted with data taken from [50]. Percentages for the digestion of macro-nutrients and the absorption of glucose, fatty acids, amino acids, and water in the three structural parts of the small intestine were adjusted following data available in [49, 59–62]. Further details of data comparison are discussed extensively in the next section.

## Results and discussion

The gastrointestinal tract is an organ system that has been studied with greater interest in the last two decades to determine the rate of glucose appearance in plasma after a meal. Most of the existing models that calculate the absorption rate of glucose in plasma are compartmental models that only consider the transit of glucose through the stomach and intestine and do not consider some physiological aspects of the digestion and absorption processes, including proteins and fats involved in glucose absorption. This section presents the results of the proposed phenomenological-based semi-physical model of the gastrointestinal tract. The simulation was carried out for the digestion, through the stomach and small intestine, of three different meals to assess the model’s prediction and description ability when compared to experimental data available in the literature [56–58]. In all three cases, the mathematical model fits the experimental data with a mean absolute error of 0.4%. Few parameters were manually calibrated due to their interpretability and the model’s basic structure based on the underlying phenomena. Additionally, the model considers many physiological aspects of the digestion and absorption of other nutrients in both the stomach and the intestine, which affect the rate of glucose appearance in plasma.

**Fig 5:**
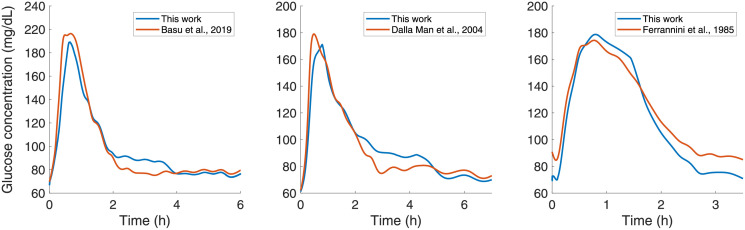
Simulated glucose concentration in portal vein following ingestion of the meals reported by [56–58], respectively.

Fig 5 shows the results of the glucose appearance rate in the portal system for the model compared with data found in the literature and their particular conditions. These data from the literature were only used to compare and evaluate the responses of the model and not to make parametric adjustments. Parametric adjustments were made with population data of percentages of digestion and absorption of nutrients in the small intestine as reported in Table 4. In [56], a novel natural tracer method is proposed to measure complex carbohydrate turnover in the postprandial state in healthy subjects. Meal content considered in this case is 52.2*g* of carbohydrates, 35.5*g* of protein, 27.1*g* of lipids, and 600.1*kcal* of the total energy content of the food. On the other hand, a reconstructed rate of glucose appearance by employing a tracer two-step method [57] is used to validate the model response following a triple-tracer mixed meal containing 10*kcal*/*kg*, 45% carbohydrates, 15% protein, and 40% fat. The rate of glucose appearance after an ingestion of glucose load in healthy subjects is also used to contrast the model response. In this case, the subjects of the study [58] ingested 1*g* of glucose per *kg* of body weight (as a 45% aqueous solution) for 5 minutes. In all three cases, the mathematical model fits the experimental data with few parameters to calibrate due to their interpretability and the model’s basic structure based on the underlying phenomena.

**Table 4:** Accumulative absorption, digestion, and inactivation percentage of substances, macronutrients, and pancreatic enzymes in the small intestine. Trypsin inactivation percentage was considered for the validation of the equivalent pancreatic protease.

Process	Substance	Duodenum		Jejunum		Ileum		Ref.
		Lit.	Sim.	Lit.	Sim.	Lit.	Sim.	
Accumulative digestion percentage	Carbohydrates	–	63.5%	75%	75.5%	84.2–89.5%	89.9%	[50]
	Proteins	33%	31.1%	60%	65.4%	89.5–94.7%	92.1%	[59, 60]
	Lipids	–	32.0%	–	44.4%	68.7–75%	72.5%	[49, 59]
Accumulative absorption percentage	Glucose	25%	25.5%	75%	75.0%	85%	85.2%	[61]
	Amino acids	30%	30.1%	80%	80.0%	90%	90.1%	[49]
	Fatty acids	30%	29.5%	85%	85.0%	100%	99.8%	[59]
	Water	14%	14.1%	44%	43.4%	83%	84.1%	[62]
Accumulative inactivation percentage of pancreatic enzymes	Pancreatic amylase	–	–	15%	15.6%	26%	26.6%	[50]
	Trypsin*	–	–	36%	37.1%	78%	74.8%	
	Pancreatic lipase	–	–	–	29.6%	75–80%	80.0%	

**Fig 6:**
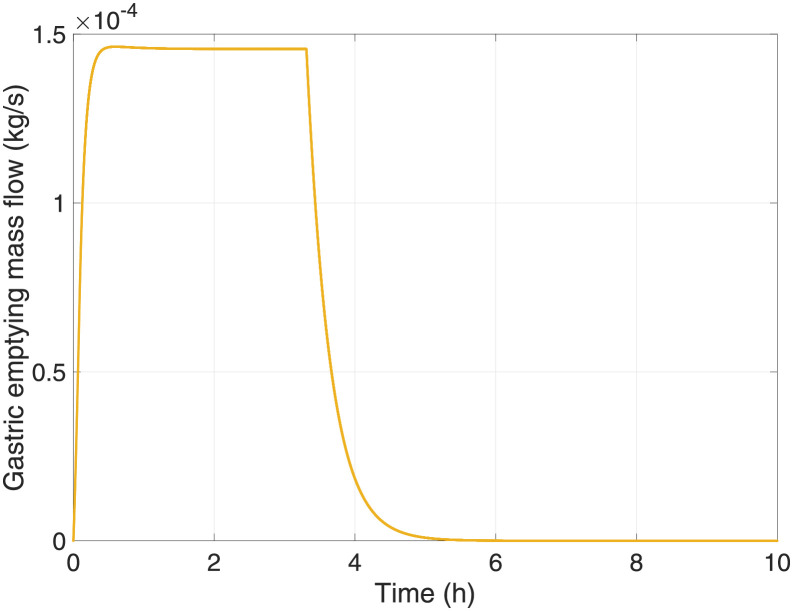
Gastric emptying of the mass flow of the chyme through the pyloric valve.

Fig 6 shows the gastric mass flow passing from the stomach to the first portion of the duodenum through the pylorus valve over time. Initially, until the chyme reaches specific rheological properties in the stomach to be emptied into the duodenum, the mass flow through the pylorus increases until it reaches a maximum point. Then, the flow remains constant for the next 2 hours, which is the duration of digestion in the stomach for the considered mixed meal, where proteins and fats are partially digested before mass flow decreases until complete emptying [63]. Digestion then continues in the small intestine, where some components are absorbed into the portal system. It is worth clarifying that the gastric emptying rate is mainly a function of the caloric density of the ingested meal and that the relationship between these variables is linear. Furthermore, the nature of the calories seems to play a minor role in determining the rate of gastric emptying in humans.

**Fig 7:**
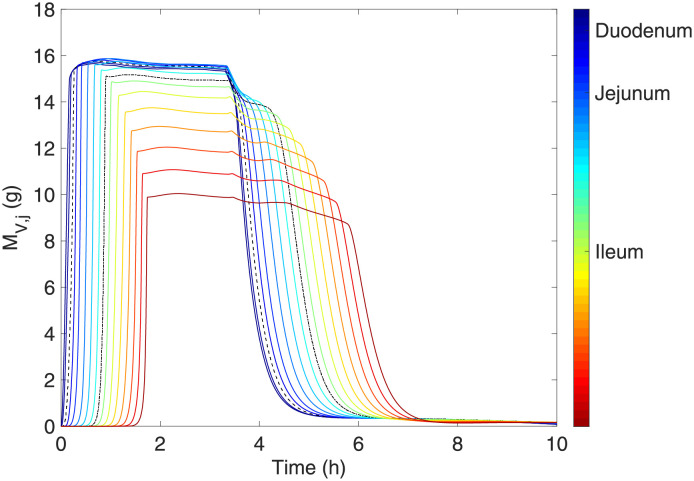
Mass contained inside each tank *j* in the postprandial period.

Fig 7 displays the accumulation of gastric mass on each portion of the small intestine in time. As can be noted, the duodenum contains the highest content of chyme. As the gastric mass is digested, nutrients are absorbed into the bloodstream, which causes a decrease in lumen food content as it moves towards the ileum until being almost completely emptied after 7 hours of digestion. This considering that there is no more ingestion during that time interval. The percentages of digestion and absorption in each part of the small intestine were obtained by adjusting the parameters for reaction kinetics and absorption rates as in the data reported in the literature.

Table 4 compares data collected from the literature (*Lit* column) with simulation data (*Sim* column) for the digestion of macronutrients, the absorption of nutrients and water, and the accumulated inactivation percentages of the considered pancreatic enzymes up to the duodenum, jejunum, and ileum. Macronutrients and their simplest, digested forms (glucose, amino acids, and fatty acids) are mostly digested and absorbed in the ileum. Undigested and non-absorbed nutrients continue to the large intestine to be fermented and converted into shorter peptides, amino acids, and fatty acids. The activities of pancreatic enzymes do not decrease in the first portion of the gut because that is where digestion begins, where pancreatic juice and bile are first mixed with the chyme. Later, activities begin decreasing during their passage from the duodenum to the terminal ileum, although the degradation rates of the individual enzymes are different [37]. While the activity of pancreatic lipase is lost more rapidly and at a higher percentage, proteases and amylase are more stable, with the activity of pancreatic amylase being the least-decreasing during intestinal digestion. Simulation data is close to the data found in the literature, indicating that the model can reliably reproduce the digestion and absorption of the nutrients in the gastrointestinal tract. Moreover, the literature has unclear percentages of digestion for carbohydrates and lipids in the duodenum and lipids in the mid-jejunum, and data available from experiments are missing. In this regard, the proposed model can provide this missing information accurately due to the high coupling of the mathematical model, the inclusion of available physiological knowledge, and the representation of the digestion and absorption processes in the human body that are as close to physiology as possible.

## Conclusions

The gastrointestinal tract is a complex organ system with many interactions between different parts, tissues, and cells. In the control of blood glucose levels, physiological aspects such as early detection of meals and rate of glucose appearance have challenged the controller’s performance in maintaining normal glucose levels and, consequently, an appropriate treatment of people with diabetes mellitus. Several studies have tried calculating the glucose appearance rate by using different techniques and mathematical modelling methodologies, commonly by compartmental models. In this study, a novel technique was used to develop a phenomenological-based semi-physical model to represent the role of the gastrointestinal tract in glucose metabolism in healthy humans. The proposed physiological model has many parameters, but their estimation was not a difficult task thanks to their interpretability. Additionally, the model is modular, meaning that, it can be used in algorithms developed to improve glucose control in people with diabetes mellitus, for other diagnostic purposes, or it can be combined with mathematical models of other organs to obtain a complete mathematical model of glucose metabolism in humans. Actually, the presented model is integrated with a liver model [64] and a model representing insulin pharmacokinetics and runs on a mobile app to predict glucose dynamics after consuming a mixed meal and assess whether carbohydrate counting improves in people who live with diabetes [65]. The model could be extended for future work by adding several functionalities such as duodenal feedback control of gastric emptying, which relies on the concentration of carbohydrate, lipid and protein digestion products, pH, osmolarity, distension and hormonal effects such as the incretin effect. Also, the chemical composition of meals and the physical nature of food remain crucial in regulating the emptying rate, affecting intestinal transit time. Consequently, modeling these phenomena is essential for understanding the relationships between the physical and chemical properties of food and digestion and absorption rates.

## Supporting information

S1 Appendix (PDF)
