# Supplementary material for: Estimation of glucose rate of appearance in portal vein circulation using a phenomenological-based model
Source: PLoS One. 2023 May 25;18(5):e0285849. doi: 10.1371/journal.pone.0285849 (PMC10212177; doi:10.1371/journal.pone.0285849)
Supplement: S1 Appendix — (PDF) [file pone.0285849.s001.pdf]

Appendix

Equations for the stomach

$$\frac{dN_I}{dt} = \dot{n}_2 - \dot{n}_8 + \sum_s (\sigma_{s,I} \cdot r_{s,I}) \tag{30}$$

$$\frac{dN_{s_I}}{dt} = x_{s_2}\ \dot{n}_2 - x_{s_8}\ \dot{n}_8 + \sigma_{s,I} \cdot r_{s,I} \tag{31}$$

$$\dot{W} = \frac{1}{\eta} (\dot{m}_I \cdot h_f) \tag{32}$$

$$\dot{m}_4 = \frac{\hat{r}_{G_{II}}}{w_{G_4}} \tag{33}$$

$$\hat{r}_{G_{II}} = \frac{1}{\Delta \bar{H}_{r_{G_{II}}}} \dot{W} \tag{34}$$

$$\frac{dM_{III}}{dt} = 0 \ \Rightarrow M_{III} = Constant \tag{35}$$

$$\frac{dw_{G_7}}{dt} = \frac{1}{M_{III}} \left(w_{G_6}\ \dot{m}_6 - w_{G_4}\ \dot{m}_4 - w_{G_7}\ \dot{m}_7 \right) \tag{36}$$

$$\dot{m}_{10,1} = \dot{m}_8 + \dot{m}_9 \tag{37}$$

$$w_{s_{10,1}} = \frac{w_{s_8} \dot{m}_8 + w_{s_9} \dot{m}_9}{\dot{m}_{10,1}} \tag{38}$$

$$\tag{39}$$

Equations for the small intestine

$$\frac{dM_{V,j}}{dt} = \dot{m}_{10,j} - \dot{m}_{11,j} - \dot{m}_{17,j} \tag{40}$$

$$\frac{dV_{V,j}}{dt} = \frac{1}{\rho_{V,j}} (\dot{m}_{10,j} - \dot{m}_{11,j} - \dot{m}_{17,j} - V_{V,j} \dot{\rho}_{V,j}) \tag{41}$$

$$\frac{dr_j}{dt} = \frac{1}{2\pi L_V r_j \rho_{V,j}} (\dot{m}_{10,j} - \dot{m}_{11,j} - \dot{m}_{17,j} - V_{V,j} \dot{\rho}_{V,j}) \tag{42}$$

$$\frac{d\rho_{V,j}}{dt} = \frac{1}{V_{V,j}} \left(\dot{m}_{10,j} - \dot{m}_{11,j} - \dot{m}_{17,j} - \rho_{V,j} \dot{V}_{V,j} \right) \tag{43}$$

$$\frac{dM_{s_{V,j}}}{dt} = w_{s_{10,j}} \dot{m}_{10,j} - \dot{n}_{s_{11,j}} \mathfrak{M}_s - w_{s_{17,j}} \dot{m}_{17,j} + V_{V,j} \mathfrak{M}_s \sum_i r_{s_{i,j}} \tag{44}$$

$$\frac{dC_{s_{V,j}}}{dt} = \frac{1}{\mathfrak{M}_s V_{V,j}} \left(w_{s_{10,j}} \dot{m}_{10,j} - \dot{n}_{s_{11,j}} \mathfrak{M}_s - w_{s_{17,j}} \dot{m}_{17,j} + V_{V,j} \mathfrak{M}_s \sum_i r_{s_{i,j}} - C_{s_{V,j}} \dot{V}_{V,j} \right) \tag{45}$$

$$\frac{dw_{s_{V,j}}}{dt} = \frac{1}{M_{V,j}} \left(w_{s_{10,j}} \dot{m}_{10,j} - \dot{n}_{s_{11,j}} \mathfrak{M}_s - w_{s_{17,j}} \dot{m}_{17,j} + V_{V,j} \mathfrak{M}_s \sum_i r_{s_{i,j}} - w_{s_{V,j}} \dot{M}_{V,j} \right) \tag{46}$$

$$\frac{dM_{VI,j}}{dt} = \dot{m}_{11,j} + \dot{m}_{14,j} - \dot{m}_{12,j} - \dot{m}_{13,j} - r_{s_{VI,j}} \tag{47}$$

$$\frac{dM_{s_{VI,j}}}{dt} = \dot{n}_{s_{11,j}} \mathfrak{M}_s + w_{s_{14,j}} \dot{m}_{14,j} - w_{s_{12,j}} \dot{m}_{12,j} - w_{s_{13,j}} \dot{m}_{13,j} - r_{s_{VI,j}} \tag{48}$$

$$\dot{m}_{16,j} = \dot{m}_{12,j} + \dot{m}_{15,j} - \dot{m}_{14,j} \tag{49}$$

$$\dot{m}_{16} = \sum_j \dot{m}_{16,j} \tag{50}$$

$$\dot{m}_{G_{16,j}} = \dot{m}_{G_{16,j}} = w_{G_{12,j}} \dot{m}_{12,j} + w_{G_{15,j}} \dot{m}_{15,j} - w_{G_{14,j}} \dot{m}_{14,j} \tag{51}$$

$$\dot{m}_{G_{16}} = \sum_j \dot{m}_{G_{16,j}} \tag{52}$$

$$w_{G_{16}} = \frac{\dot{m}_{G_{16}}}{\dot{m}_{16}} \tag{53}$$

$$\dot{m}_{17,j} = \dot{m}_{10,j+1} \tag{54}$$

$$w_{s_{17,j}} = w_{s_{10,j+1}} \tag{55}$$

$$\dot{m}_{10,j+1} = c_{v_j}\ \rho_{V,j} \sqrt{P_{V,j} - P_{V,j+1}} \tag{56}$$

$$\tag{57}$$

Extended structure of the model

Table 5. Constitutive and assessment equations for structural parameters of the model.

#	Description	Cons./Ass. Equation	Inst.	Ref.
1	Molar flow of gastric juices entering the stomach through the stomach walls.	$\dot{n}_2 = \frac{\dot{m}_2}{\mathfrak{M}_{GJ}}$	1	M
2	Molar flow of gastric mass leaving the stomach due to gastric emptying.	$\dot{n}_8 = \frac{\dot{m}_8}{\mathfrak{M}_I}$	1	M
3	Rate of reaction for the substance s: Lip, Pro, GL, GP.	$r_{s,I} = \begin{cases} k_{0_{Lip_I}} \cdot C_{Lip_I}^{n_I} \cdot C_{GL_I}^{m_I} \cdot e^{\frac{-E_{a_{Lip_I}}}{R \cdot T}} & \text{if } s = Lip \\ k_{0_{Pro_I}} \cdot C_{Pro_I}^{n_I} \cdot C_{GP_I}^{m_I} \cdot e^{\frac{-E_{a_{Pro_I}}}{R \cdot T}} & \text{if } s = Pro \\ k_{0_{GL_I}} \cdot C_{GL_I}^{m_I} \cdot e^{\frac{-E_{a_{GL_I}}}{R \cdot T}} & \text{if } s = GL \\ k_{0_{GP_I}} \cdot C_{GP_I}^{m_I} \cdot e^{\frac{-E_{a_{GP_I}}}{R \cdot T}} & \text{if } s = GP \end{cases}$	4	[1]
4	Molar fraction of substance s in the stream containing gastric juices.	$x_{s_2} = \begin{cases} 1 & \text{if } s = GJ \\ 0 & \text{if } s \neq GJ \end{cases}$	1	A
5	Molar fraction of each substance s (CHO, Lip, Pro, G, FA, AA, W, Fib, Gly, GL, GP, GJ) in the stomach emptying into the duodenum.	$x_{s_8} = \frac{N_{s_I}}{N_I}$	12	[1]
6	Efficiency of the stomach wall to perform mechanical work.	$\eta = 0.5$	1	A
7	Mass flow of gastric mass that churns within the stomach.	$\dot{m}_I = \dot{V}_I \cdot \rho_I$	1	M
8	Friction losses experienced by the mass flow when circulating in the piping circuit (stomach).	$h_f = \sum_s \frac{K_{sc} \cdot v_{sc}^2}{2}$	1	[2]
9	Mass fraction of glucose in the stream containing the required reactants	$w_{G_4} = 0.484$	1	M
10	Specific molar heat of reaction of glucose combustion.	$\Delta \bar{H}_{r_{G_{II}}} = 2813\, kJ \cdot mol^{-1}$	1	[3]
11	Mass fraction of glucose that supplies the stomach wall through the artery.	$w_{G_6} = \frac{C_{G_6}}{\rho_b}$	1	M
12	Mass flow of blood supplying the stomach through the artery.	$\dot{m}_6 = \dot{V}_6 \cdot \rho_b$	1	[1]
13	Mass flow of blood from the stomach wall that drains through the vein.	$\dot{m}_7 = \dot{m}_6$	1	M
14	Mass flow resulting from mixing ingested food emptied from the stomach.	$\dot{m}_8 = \begin{cases} \text{if } V_I(t) > 0.35 V_I(0): \\ \dot{m}_{8(i-1)} + \frac{1}{\tau_{ini}} \left(\dot{m}_{8req} - \dot{m}_{8(i-1)} \right) t_s \\ \text{if } V_I(t) \leq 0.35 V_I(0): \\ \dot{m}_{8(i-1)} + \frac{1}{\tau_{end}} \left(\dot{m}_{8req} - \dot{m}_{8(i-1)} \right) t_s \end{cases}$	1	A
15	Mass flow of bile and pancreatic juice entering the duodenum.	$\dot{m}_9 = \rho_9 \cdot SP \left(\dot{V}_{bile,basal} + 3\, \dot{V}_{PJ,basal} \right)$	1	A

Abbreviations. A: Assumed, I: Identified, M: From the model.

(This table continues on the next page)

(It comes from the Table 5 above)

#	Description	Cons./Ass. Equation	Inst.	Ref.
16	Mass fraction of component s (CHO, Pro, Lip, G, AA, FA, W, F, GL, GP, GJ, Gly) of ingested food that are emptied into the duodenum.	$w_{s_8} = x_{s_I} \cdot \frac{1}{\mathfrak{M}_I} \cdot \mathfrak{M}_s$	12	M
17	Mass fraction of s (PA, PP, PL) in pancreatic juices and bile emptied into the duodenum.	$w_{s_9} = \frac{C_{s_9} \mathfrak{M}_s}{\rho_9}$	3	M
18	Mass flow of gastric mass entering a tank (portion j of the intestine).	$\dot{m}_{10,j} = \sum_s \dot{m}_{s_{10,j}}$	1	M
19	Mass absorption rate through each portion j of the intestinal wall.	$\dot{m}_{11,j} = \sum_s \dot{n}_{s_{11,j}} \cdot \mathfrak{M}_s$	1	M
20	Length of an elastic tank.	$L_V = \frac{L}{n}$	1	A
21	Mass fraction of s (CHO, G, Pro, AA, Lip, FA, W, F, GJ, GL, GP, Gly, CHO*, Pro*, PA, PP, PL, PA-In, PP-In, PL-In) in the stream 10 entering the j-th tank.	$w_{s_{10,j}} = \frac{\dot{m}_{s_{10,j}}}{\dot{m}_{10,j}}$	20	[1]
22	Term for the molar rate of absorption of s (G, AA, FA, W) in the j-th tank ($z : Duo, Je, Ile$).	$\dot{n}_{s_{11,j}} = \begin{cases} \left(K_{d_{G,z}} + \frac{vmax_{G,z}\, C_{G_{V,j}}}{K_{M_G} + C_{G_{V,j}}}\right) M_{VI,dry} \text{ if } s = G \\ \left(\frac{vmax_{AA,z}\, C_{AA_{V,j}}}{K_{M_{AA}} + C_{AA_{V,j}}}\right) L_V \text{ if } s = AA \\ A_M K_{FA,z} \left(C_{FA_{V,j}} - C^*_{FA_{VI}}\right) \text{ if } s = FA \\ L_V \left(K_{W,z}\, \dot{m}_{10,1} + 2.67e-7\right) \text{ if } s = W \end{cases}$	4	[4]<br>[5]<br>A<br>A
23	Molar rate of reaction of the component s (CHO, G, W, PA, AA, Pro, PP, FA, Lip, Gly, PL, PA-In, PL-In, PP-In) in the reaction i taking place in the small intestine.	$r_{s_{i,j}}$ = See Table 3	16	
24	Mass flow of absorbed components passing into the blood in capillaries.	$\dot{m}_{12,j} = \sum_s \dot{n}_{s_{11,j}} \cdot \mathfrak{M}_s$ for $s : W, G, AA$	1	M
25	Mass flow of components drained by the lymph.	$\dot{m}_{13,j} = \sum_s \dot{n}_{s_{11,j}} \cdot \mathfrak{M}_s$ for $s : FA, Gly$	1	M
26	Mass flow of glucose for cell uptake of the small intestine wall.	$\dot{m}_{14,j} = \begin{cases} 0 & \text{if } M_{G_{V,j}} \neq 0 \\ 1.8e-8 & \text{if } M_{G_{V,j}} = 0 \end{cases}$	1	A<br>I
27	Mass flow of blood supplying the small intestine.	$\dot{m}_{15,j} = \rho_b \cdot \dot{V}_{15}$	1	M
28	Rate of consumption of s (available in the intestinal lumen) by each portion j of the small intestine wall.	$r_{G_{VI,j}} = \begin{cases} 0.1\, \mathfrak{M}_G\, \dot{n}_{G_{11,j}} & \textit{if } M_{G_{V,j}} \neq 0 \\ 1.8e-8 & \textit{if } M_{G_{V,j}} = 0 \end{cases}$	1	[6]
29	Mass fractions of s (G, AA, W) that are absorbed through stream 12.	$w_{s_{12,j}} = \frac{\dot{m}_{11,j}}{\dot{m}_{12,j}}$	3	M
30	Mass fractions of s (FA, Gly) that are exchanged with the interstitium through stream 13.	$w_{s_{13,j}} = \frac{\dot{m}_{11,j}}{\dot{m}_{13,j}}$	2	M
31	Mass fractions of s (G) in the stream 14.	$w_{s_{14,j}} = \begin{cases} 0 \text{ for } s \neq G \\ 1 \text{ for } s = G \end{cases}$	1	M
32	Mass fraction of glucose via arterial blood irrigating the small intestine.	$w_{G_{15,j}}$= Data from the literature	1	[7]
33	Hydrodynamic pressure on gastric mass flow entering the portion j.	$P_{V,j}$= See Eq 29	1	[8]
34	Valve flow coefficient.	$c_{v_j} = f_{x_j}\, C_v$	1	[9]

Abbreviations. A: Assumed, I: Identified, M: From the model.

Table 6. Constitutive equations for functional parameters of the gastrointestinal model.

#	Description	Cons./Ass. Equation	Inst.	Ref.
1	Mass flow of gastric juices entering the stomach through the stomach wall.	$\dot{m}_2 = \dot{V}_2 \cdot \rho_{GJ}$	1	M
2	Molecular mass of the gastric mixture contained in the stomach.	$\mathfrak{M}_I = \sum_s x_{s_I} \cdot \mathfrak{M}_s$	1	M
3	Molar-volumetric concentration of component s (Lip, Pro, GL, GP), after the addition of gastric juices but before the enzymatic reactions and emptying.	$C_{s_I} = \frac{N_{s_I}}{V_{I_{sv}}}$	4	M
4	Volumetric flow of gastric mass within the stomach.	$\dot{V}_I = v_{0_{I_6}} \cdot A_{flow_{I_6}}$	1	M
5	Density of gastric mass in the stomach.	$\rho_I = \sum_s \frac{1}{w_{s_I}/\rho_s}$	1	[1]
6	Friction factor for each section of the pipe circuit representing the stomach. Total number of sections: 12	$K_{sc} = \begin{cases} K_{straight} = f_{Darcy} \frac{L_{straight_{sc}}}{D_{sc}} \\ K_{expan} = [1 - \frac{SD}{HD}^2]^2 \\ K_{cont} = 0.5[1 - \frac{SD}{HD}^2]^2 \\ K_{180^\circ_{Elbow}} = \frac{1000}{Re_{sc}} + 0.6(1 + \frac{1}{ID}) \end{cases}$	12	[10]
7	Velocity of the gastric mass in each section of the pipe circuit representing the stomach.	$v_{sc} = \frac{\dot{V}_I}{A_{flow_{I_{sc}}}}$	12	M
8	Characteristic time of gastric emptying start.	$\tau_{ini} = 0.5 \cdot t_{lag}$	1	A
9	Mass flow out of the stomach required for the flow to be isocaloric.	$\dot{m}_{8_{req}} = \frac{\dot{E}_{8_{iso}}}{e_{meal} w_{8_{meal}}}$	1	M
10	Molar fraction of component s (Lip, AG, Pro, AA, CHO, G, F, W, GJ, GL, GP, Gly) in the stomach.	$x_{s_I} = \frac{N_{s_I}}{N_I}$	12	M
11	Mass flow of component s (CHO, G, Pro, AA, Lip, FA, PA, PP, PL, GP, GL, W, Gly, AP-In, PP-In, PL-In, F, GJ) entering each portion j of the small intestine.	$\dot{m}_{s_{10,j}} = \dot{m}_{17,j} \cdot w_{s_{V,j}}$	18	M
12	Length of the small intestine.	$L = \sum_z L_z$	1	M
13	Dry mass of a portion j of intestinal wall.	$M_{VI,dry} = \rho_{VI,dry} \cdot V_{VI}$	1	M
14	Concentration of s (PA, PP, PL) in bile and pancreatic juice flow.	$C_{s_9} = \begin{cases} \left(\frac{SP}{SP_{max}}\right)\left(\frac{Act_{PA_9}}{\widehat{Act}_{PA_9}\mathfrak{M}_{PA}}\right) & \text{if } s: PA \\ C_{PA_9}\left(\frac{Z_{P\to A}}{\widehat{Act}_{PP_9}\mathfrak{M}_{PP}}\right) & \text{if } s: PP \\ C_{PA_9}\left(\frac{Z_{L\to A}}{\widehat{Act}_{PL_9}\mathfrak{M}_{PL}}\right) & \text{if } s: PL \end{cases}$	3	M
15	Pancreatic amylase activity.	$Act_{AP_9} = 0.101 + \frac{0.05059\, C_{FA_{V,1}}}{C_{FA_{V,1}} + 0.5321}$	1	[11]
16	Effective area for the mass transfer of fatty acids in the enterocytes.	$A_M = 2 \cdot \pi \cdot \bar{r} \cdot L_V \cdot F_{fold}$	1	A
17	Fung material constant C for the small intestine wall of tank j.	$C_j = -12.0\, L_j^4 + 165.9\, L_j^3 - 782.1\, L_j^2 + 1317\, L_j + 1256$	1	[12]

Abbreviations. A: Assumed, I: Identified, M: From the model.

(This table continues on the next page)

(It comes from the Table 6 above)

#	Description	Cons./Ass. Equation	Inst.	Ref.
18	Fung material constant a_1 for the small intestine wall of tank j.	$a_{1_j} = -1.61\, L_j^3 + 16.8\, L_j^2 - 44.7\, L_j + 72.70$	1	[12]
19	Fung material constant a_2 for the small intestine wall of tank j.	$a_{2_j} = 1.5\, L_j^3 - 17.4\, L_j^2 + 51.5\, L_j + 29.02$	1	[12]
20	Cross-area of the tank j.	$A_{V,j} = \pi r_j^2$	1	M
21	Cross-area of the tank j when deflated.	$A_{0_{V,j}} = \pi r_{0_j}^2$	1	M
22	Cross area of the intestinal wall of tank j.	$A_{wall_{V,j}} = \pi \left(r_{0_j} + h_{0_j} \right) - A_{0_{V,j}}$	1	M
23	Volumetric flow of gastric juices entering the stomach.	$\dot{V}_2 = \frac{k_{\dot{V}_2}}{t_{lag}} \cdot V_I$	1	M
24	Total volume in the stomach, before emptying, including gastric juices.	$V_{I_{sv}} = V_I + (\dot{V}_2 \cdot t_s)$	1	A
25	Cross area of each section sc.	$A_{flow_{I_6}} = FC_A\, A_{0_{flow_{I_{sc}}}}$	12	A
26	Mass fraction of component s in the stomach.	$w_{s_I} = \frac{M_{s_I}}{M_I}$	11	M
27	Darcy factor.	$f_{Darcy} = \frac{64}{Re_{sc}}$	1	[1]
28	Reynolds number for straight and elbow sections (sc=1, 3, 5, 6, 10, 12).	$Re_{sc} = \frac{\rho_I \cdot v_{sc} \cdot D_{sc}}{\mu_I}$	6	M
29	Cross sectional area of the fitting at the distal extreme (in the case of contractions and expansions, the smallest SD diameter is considered).	$A_{0_{flow_{I_{sc}}}} = \pi \frac{D_{sc}^2}{4}$	12	M
30	Specific energy content of the ingested food mass.	$e_{meal} = \frac{E_{meal}}{\sum_s M_{s_I}}$	1	M
31	Mass fraction of caloric food ingested (s: Lip, FA, Pro, AA, CHO, G).	$w_{8_{meal}} = \sum_s w_{s_I}$	1	M
32	Volume of a portion j of intestinal wall.	$V_{VI} = 2\pi r_{0_j} h_{0_j} L_V$	1	M
33	Gastric emptying mass flow of component s (CHO, G, Pro, AA, Lip, FA, W, F, GJ, GL, GP, PA, PP, PL, Gly, PA-In, PP-In, PL-In).	$\dot{m}_{s_{10,1}} = \dot{n}_8 \cdot x_{s_I} \cdot \mathfrak{M}_s + w_{s_9} \cdot \dot{m}_9$	18	M
34	Valve displacement following the inherent valve characteristic.	$f_{x_j} = \frac{P_{V,j} - P_{V,j+1}}{\bar{P}_{cont}}$	1	A
35	Equivalent mean contraction pressure	$\bar{P}_{cont} = DC \cdot P_{cont}$	1	M
36	Duty cycle.	$DC = \frac{\tau}{t_{pw}}$	1	M
37	Volume of gastric mass present in the stomach.	$V_I = \sum_s \frac{N_{s_I} \cdot \mathfrak{M}_s}{\rho_s}$	1	M
38	Correction factor for the area of each stomach section.	$FC_A = \frac{V_I}{A_{0_{flow_{I_{sc}}}} \cdot L_{I_{sc}}}$	12	A
39	Total mass in the stomach.	$M_I = \sum_s M_{s_I}$	1	M
40	Viscosity of the gastric mass.	$\mu_I = 0.001 \cdot (\mu_{I_{MP}} + \mu_{I_{limit}})$	1	[13]
41	Energy content of the initial food.	$E_{meal} = (M_{Lip_I} + M_{FA_I}) \cdot E_{Lip} + (M_{Pro_I} + M_{AA_I}) \cdot E_{Pro} + (M_{CHO_I} + M_{G_I}) \cdot E_{CHO}$	1	M

Abbreviations. A: Assumed, I: Identified, M: From the model.

(This table continues on the next page)

(It comes from the Table 6 above)

#	Description	Cons./Ass. Equation	Inst.	Ref.
42	Basal radius of the small intestine.	$r_{0_j} = -0.081\, L_j^2 + 0.15\, L_j + 6.21$	1	[14]
43	Thickness of the intestinal wall.	$h_{0_j} = -0.0015\, L_j^3 + 0.014\, L_j^2 - 0.032\, L_j + 1.53$	1	[14]
44	Polynomial correlation for a mix meal.	$\mu_{I_{MP}} = -4346.483214 \cdot FVM^4 + 5855.694121 \cdot FVM^3 - 1767.803009 \cdot FVM^2 + 295.563994 \cdot FVM - 10.04508874$	1	[13]
45	Length traveled by gastric mass in the tank j.	$L_j = j \cdot L_V$	1	M
46	Modified Viscosity Factor.	$FVM = FD^{2.97}$	1	see [15]
47	Dilution factor.	$FD = -6.007024928 \times 10^{-8} \cdot t_{Sim}^3 + 7.018107705 \times 10^{-6} \cdot t_{Sim}^2 - 0.002301353528 \cdot t_{Sim} + 0.6412266547$	1	[16, 17, 18]

Abbreviations. A: Assumed, I: Identified, M: From the model.

Table 7. Assessment equations and constants of the gastrointestinal model.

Symbol	Description	Value	Inst.	Ref.
$\sigma_{s,I}$	Stoichiometric coefficient of component s: Lip, FA, W, Gly, Pro, AA, GL, GP, in the reactions of digestion that takes place in the stomach.	$\sigma_{(Lip,Pro),I} = -1$ $\sigma_{(LG,PG),I} = -1$ $\sigma_{FA,I} = 3$ $\sigma_{W,I} = -3$ $\sigma_{Gly,I} = 1$ $\sigma_{AA,I} = 153$	8	M
$\mathfrak{M}_s$	Molar mass of the component s (Lip, Pro, CHO, FA, AA, G, Fib, W, GJ, GL, GP, Gly, PA, PP, PL, PA-In, PP-In, PL-In).	-	18	[19]
$k_{0_{s_I}}$	Rate constant due to the frequency of molecular collisions in the correct orientation for component s (Lip, Pro, GL, GP) in the stomach.	See Table 4 in [15]	4	I
$E_{a_{s_I}}$	Activation energy for reaction of component s (Lip, Pro, GL, GP) in the stomach.	See Table 4 in [15]	4	I
n_I	Exponent for the concentration of lipids and proteins in their degradation reactions in the stomach.	1	1	
m_I	Exponent for the concentration of lipases and proteases in their degradation reactions in the stomach.	1	1	
C_{G_6}	Glucose concentration in the arterial blood supplying the stomach.	$0.9\, kg \cdot m^{-3}$	1	A
ρ_b	Blood density.	$1060\, kg \cdot m^{-3}$	1	[20]
$\dot{V}_6$	Volumetric flow of arterial blood supplying the stomach	$0.00001\, m^3 \cdot s^{-1}$	1	
τ_{end}	Characteristic time for the end of gastric emptying.	$1200s$	1	A
t_s	Sampling time.	$1s$	1	A
$\dot{V}_{bile,basal}$	Basal bile volume flow.	$8.33 \times 10^{-9}\, m^3 \cdot s^{-1}$	1	[21,22]
$\dot{V}_{PJ,basal}$	Basal pancreatic juice volume flow.	$4.17 \times 10^{-9}\, m^3 \cdot s^{-1}$	1	[23]
ρ_9	Density of bile and pancreatic juice.	$1000\, kg \cdot m^{-3}$	1	A
SP	Basal percentage of bile and pancreatic juice secretion	*Determined by the fuzzy model [24].	1	[25]
SP_{max}	Maximum basal secretion percentage.	500%	1	A
$\widehat{Act}_{Ez_9}$	Specific activity of pancreatic enzymes (Ez): pancreatic amylase (PA), pancreatic protease considered here trypsin as equivalent (PP), and pancreatic lipase (PL).	$\widehat{Act}_{PA,PL_9} = 1.2 \times 10^{-2}\, kU \cdot mg^{-1}$ $\widehat{Act}_{PP_9} = 7.73 \times 10^{-3}\, kU \cdot mg^{-1}$	3	[25, 26]
$Z_{P \to A}$	Ratio of protease to amylase.	0.54	1	[25]
$Z_{L \to A}$	Ratio of lipase to amylase	3.5	1	[25]
L_z	Length of each part of small intestine (z: Duodenum, jejunum, and ileum).	$Duo = 0.2\, m$ $Je = 2.3\, m$ $Ile = 2.7\, m$	3	[27]
$K_{d_{G,z}}$	Mass transfer constant of passive glucose absorption in each section of small intestine (z: duodenum, jejunum, and ileum).	$Duo = 1.955 \times 10^{-5}\, m^3 \cdot kg^{-1} \cdot s^{-1}$ $Je = 4.301 \times 10^{-6}\, m^3 \cdot kg^{-1} \cdot s^{-1}$ $Ile = 1.251 \times 10^{-8}\, m^3 \cdot kg^{-1} \cdot s^{-1}$	3	[4]

Abbreviations. A: Assumed, I: Identified, M: From the model.

(This table continues on the next page)

(It comes from the Table 7 above)

Symbol	Description	Value	Inst.	Ref.
$v_{max_{G,z}}$	Maximum velocity of saturable glucose absorption in each section z of intestine (z: Duodenum, jejunum, and ileum).	$Duo = 1.667 \times 10^{-10}\, m^3 \cdot kg^{-1} \cdot s^{-1}$<br>$Je = 3.667 \times 10^{-11}\, m^3 \cdot kg^{-1} \cdot s^{-1}$<br>$Ile = 1.067 \times 10^{-13}\, m^3 \cdot kg^{-1} \cdot s^{-1}$	3	[4]
K_{M_G}	Michaelis constant of glucose absorption.	$2.06 \times 10^{-3}\, kmol \cdot m^{-3}$	1	[4]
$v_{max_{AA,z}}$	Maximum velocity of saturable amino acid absorption in each section z of intestine (z: Duodenum, jejunum, and ileum).	$Duo = 1.222 \times 10^{-6}\, kmol \cdot kg^{-1} \cdot s^{-1}$<br>$Je = 1.000 \times 10^{-8}\, kmol \cdot kg^{-1} \cdot s^{-1}$<br>$Ile = 1.889 \times 10^{-9}\, kmol \cdot kg^{-1} \cdot s^{-1}$	3	[5]
$K_{M_{AA}}$	Michaelis constant of amino acid absorption.	$3.60 \times 10^{-2}\, kmol \cdot m^{-3}$	1	I
$K_{FA,z}$	Fatty acid mass transfer coefficient.	$Duo = 1.945 \times 10^{-7}\, m \cdot s^{-1}$<br>$Je = 2.123 \times 10^{-8}\, m \cdot s^{-1}$<br>$Ile = 2.805 \times 10^{-9}\, m \cdot s^{-1}$	3	[5]
$C^*_{FA_{VI}}$	Fatty acid equilibrium concentration.	$1 \times 10^{-4}\, kmol \cdot m^{-3}$	1	A
$K_{W,z}$	Water mass transfer coefficient.	$Duo = 1.05 \times 10^{-3}\, kmol \cdot kg^{-1} \cdot m^{-1}$<br>$Je = 2.32 \times 10^{-4}\, kmol \cdot kg^{-1} \cdot m^{-1}$<br>$Ile = 1.31 \times 10^{-3}\, kmol \cdot kg^{-1} \cdot m^{-1}$	3	I
$\sigma_{s,1-6}$	Stoichiometric coefficient of s (W, CHO, PA, Pro, PP, Lip, PL) in the reactions of digestion that takes place in the small intestine[a].	-1	7	M
$\sigma_{s,1-6}$	Stoichiometric coefficient of s (G, CHO*, PA-In, AA, Pro*, PP-In, Gly, PL-In) in the reactions of digestion that takes place in the small intestine[b].	1	8	M
$\sigma_{s,5}$	Stoichiometric coefficient of water and fatty acids in reaction 5 taking place in the small intestine to digest fat.	$s: W = -3$<br>$s: FA = 3$	1	M
$k_{0_{1,z}}$	Pre-exponential factor of carbohydrate digestion in each section z (duodenum, jejunum, ileum) of intestine.	$k_{0_{1,Duo}} = 8.31 \times 10^9\, s^{-1}$<br>$k_{0_{1,Je}} = 6.89 \times 10^8\, s^{-1}$<br>$k_{0_{1,Ile}} = 5.54 \times 10^7\, s^{-1}$	3	I
E_{a_1}	Activation energy of carbohydrate digestion in the intestine.	$5.749 \times 10^7\, J \cdot kmol^{-1}$	1	[28]
$k_{0_{2,z}}$	Pre-exponential factor of pancreatic amylase inactivation in each section z (duodenum, jejunum, ileum) of intestine.	$k_{0_{2,Duo}} = 2.88 \times 10^{-3}\, s^{-1}$<br>$k_{0_{2,Je}} = 4.80 \times 10^{-4}\, s^{-1}$<br>$k_{0_{2,Ile}} = 4.80 \times 10^{-4}\, s^{-1}$	3	[25]
E_{a_2}	Activation energy of pancreatic amylase inactivation in the intestine.	$2.54 \times 10^7\, J \cdot kmol^{-1}$	1	[29]
$k_{deg_{2,z}}$	Denaturation rate constants of pancreatic amylase inactivation.	$k_{deg_{2,Duo}} = 2.01 \times 10^{-4}\, s^{-1}$<br>$k_{deg_{2,Je}} = 3.34 \times 10^{-5}\, s^{-1}$<br>$k_{deg_{2,Ile}} = 3.34 \times 10^{-5}\, s^{-1}$	3	I

[a] In the reaction 5, the stoichiometric coefficient for water (W) is −3.

[b] In the reaction 5, the stoichiometric coefficient for fatty acids (FA) is 3.

Abbreviations. A: Assumed, I: Identified, M: From the model.

(This table continues on the next page)

(It comes from the Table 7 above)

Symbol	Description	Value	Inst.	Ref.
$k_{0_{3,z}}$	Pre-exponential factor of protein digestion in each section z (duodenum, jejunum, ileum) of intestine.	$k_{0_{3,Duo}} = 1.58 \times 10^{15}\, s^{-1}$ $k_{0_{3,Je}} = 7.88 \times 10^{15}\, s^{-1}$ $k_{0_{3,Ile}} = 1.97 \times 10^{14}\, s^{-1}$	3	I
E_{a_3}	Activation energy of protein digestion in the intestine.	$8.95 \times 10^7\, J \cdot kmol^{-1}$	1	[30]
$k_{0_{4,z}}$	Pre-exponential factor of pancreatic protease inactivation in each section z (duodenum, jejunum, ileum) of intestine.	$k_{0_{4,Duo}} = 2.46 \times 10^{11}\, s^{-1}$ $k_{0_{4,Je}} = 3.42 \times 10^{11}\, s^{-1}$ $k_{0_{4,Ile}} = 2.05 \times 10^{11}\, s^{-1}$	3	I
E_{a_4}	Activation energy of pancreatic protease inactivation in the intestine.	$8.95 \times 10^7\, J \cdot kmol^{-1}$	1	[30]
$k_{0_{5,z}}$	Pre-exponential factor of lipid digestion in each section z (duodenum, jejunum, ileum) of intestine.	$k_{0_{5,Duo}} = 7.06 \times 10^9\, s^{-1}$ $k_{0_{5,Je}} = 9.28 \times 10^7\, s^{-1}$ $k_{0_{5,Ile}} = 7.66 \times 10^7\, s^{-1}$	3	I
E_{a_5}	Activation energy of lipid digestion in the intestine.	$5.98 \times 10^7\, J \cdot kmol^{-1}$	1	[31]
$k_{0_{6,z}}$	Pre-exponential factor of pancreatic lipase inactivation in each section z (duodenum, jejunum, ileum) of intestine.	$k_{0_{6,Duo}} = 2.95 \times 10^{11}\, s^{-1}$ $k_{0_{6,Je}} = 2.95 \times 10^{11}\, s^{-1}$ $k_{0_{6,Ile}} = 4.43 \times 10^{11}\, s^{-1}$	3	I
E_{a_6}	Activation energy of pancreatic lipase inactivation in the intestine.	$1.063 \times 10^8\, J \cdot kmol^{-1}$	1	[29]
$k_{deg_{6,z}}$	Denaturation rate constants of pancreatic lipase inactivation.	$k_{deg_{6,Duo}} = 4.62 \times 10^{-4}\, s^{-1}$ $k_{deg_{6,Je}} = 4.62 \times 10^{-4}\, s^{-1}$ $k_{deg_{6,Ile}} = 6.92 \times 10^{-4}\, s^{-1}$	3	I
R	Universal ideal gas constant.	$8317.4\, J \cdot kmol^{-1} \cdot K^{-1}$	1	[9]
T	Body temperature.	$310.15\, K$	1	A
E_{zz}	Axial strain of the small intestine.	0.1	1	[12]
$\dot{V}_{15}$	Volumetric blood flow supplying the small intestine.	$10.6\, dL \cdot m^{-1}$	1	[20]
$v_{0_{I_6}}$	Stirring speed in the lower curvature of the smallest diameter of the stomach (section 6).	$0.105\, m \cdot s^{-1}$	1	
$L_{straight_{sc}}$	Length of each straight sections $sc: 1, 3, 5, 10$ of the pipe circuit.	See Table 5 in [15]	4	
D_{sc}	Internal diameter of each straight sections $sc: 1, 3, 5, 10$ of the pipe circuit.	See Table 5 in [15]	4	
SD	Smaller diameter in contraction/expansion sections $sc: 2, 4, 7, 8, 9, 11$ of the pipe circuit.	See Table 5 in [15]	6	
HD	Higher diameter in contraction/expansion sections $sc: 2, 4, 7, 8, 9, 11$ of the pipe circuit.	See Table 5 in [15]	6	
ID	Internal diameter in elbow sections $sc: 6, 12$ of the pipe circuit.	See Table 5 in [15]	2	
t_{lag}	Lag time for dilution to stop.	$111\, m$	1	I

Abbreviations. A: Assumed, I: Identified, M: From the model.

(This table continues on the next page)

(It comes from the Table 7 above)

Symbol	Description	Value	Inst.	Ref.
$\dot{E}_{8_{iso}}$	Energy flow out of the stomach.	$3\,kcal \cdot m^{-1}$	1	[32]
$\rho_{VI,dry}$	Density of dry small intestine.	$1400\,kg \cdot m^{-3}$	1	[33]
$\bar{r}$	Mean overall small intestine radius.	$0.02\,m$	1	[32]
F_{fold}	Small intestine fold change.	7.22	1	[34]
$k_{\dot{V}_2}$	Constant of proportionality for the addition of gastric juices.	2	1	A
C_v	Maximum valve flow coefficient with a pressure drop of 60°.	$1 \times 10^{-9}\,m^3 \cdot s^{-1} \cdot Pa^{-0.5}$	1	I
P_{cont}	Pressure exerted by periodic small intestine wall contractions on the chyme flow for the postprandial state.	$2666\,Pa$	1	[35]
τ	Duration of muscle contraction during an average peristaltic wave.	$3.1\,s$	1	[35, 36]
t_{pw}	Period of occurrence of an average peristaltic wave.	$25s$	1	[35, 36]
$L_{I_{sc}}$	Length of each section sc of the pipe circuit.	$L_{I_1} = 2.3\,cm$ $L_{I_5} = 2.2\,cm$ $L_{I_9} = 3.2\,cm$<br>$L_{I_2} = 3.2\,cm$ $L_{I_6} = 3.6\,cm$ $L_{I_{10}} = 3.3\,cm$<br>$L_{I_3} = 2.2\,cm$ $L_{I_7} = 1.4\,cm$ $L_{I_{11}} = 3.1\,cm$<br>$L_{I_4} = 1.6\,cm$ $L_{I_8} = 2.7\,cm$ $L_{I_{12}} = 4.9\,cm$	12	[15]
$\mu_{I_{limit}}$	Lower limit of the viscosity of the mixture.	10P	1	
E_s	Energy content of macronutrients (s: Lip, Pro, CHO).	9.19, 5.5, $4.1\,kcal \cdot g^{-1}$	3	[37]
M_{s_I}	Grams of food ingested (s: Lip, Pro, CHO, FA, AA, G).	$M_{Lip} = 27g$ $M_{Pro} = 35g$<br>$M_{CHO} = 52g$ $M_{FA,AA,G} = 0g$<br>$M_W = 457.29g$ $M_F = 5g$	8	A
ρ_s	Density of component s in the stomach: Lip, Pro, CHO, G, Fib, W, GJ, AA, FA, Gly, GL, GP.	$-\,kg \cdot m^{-3}$	12	
n	Number of tanks in which the intestine is partitioned.	52	1	A
t_{Sim}	Simulation time.	10h	1	M

Abbreviations. A: Assumed, I: Identified, M: From the model.

References

1. Perry, Robert H. and Green, Don W. Perry's Chemical Engineers' Handbook. 8th ed. McGraw-Hill Professional; 2008:2400.

2. Darby, Ron. Chemical engineering fluid mechanics. New York: Marcel Dekker; 2001.

3. Kuriyan, John and Konforti, Boyana and Wemmer, David. The molecules of life: Physical and chemical principles. WW Norton & Company; 2012

4. Dawson, D. J. and Burrows, P. C. and Lobley, R. W. and Holmes, R. The kinetics of monosaccharide absorption by human jejunal biopsies: Evidence for active and passive processes. Digestion. 1987;38(2): 124–132. doi: 10.1159/000199581

5. Fleshler, B. and Butt, J. H. and Wismar, J. D. Absorption of glycine and L-alanine by the human jejunum. The Journal of Clinical Investigation. 1966;45(9): 1433–1441. doi: 10.1172/JCI105451

6. Yang, Huansheng and Wang, Xiaocheng and Xiong, Xia and Yin, Yulong. Energy metabolism in intestinal epithelial cells during maturation along the crypt-villus axis. Scientific Reports. 2016; 6: 1-13. doi: 10.1038/srep31917

7. Basu, Rita and Schiavon, Michele and Petterson, Xuan Mai and Hinshaw, Ling and Slama, Michael and Carter, Rickey and Man, Chiara Dalla and Cobelli, Claudio and Basu, Ananda. A novel natural tracer method to measure complex carbohydrate metabolism. American Journal of Physiology - Endocrinology and Metabolism. 2019;317(3):E483–E493. doi: 10.1152/AJPENDO.00133.2019

8. Ma, Yinji and Choi, Jungil and Hourlier-Fargette, Aurélie and Xue, Yeguang and Chung, Ha Uk and Lee, Jong Yoon and Wang, Xiufeng and Xie, Zhaoqian and Kang, Daeshik and Wang, Heling and Han, Seungyong and Kang, Seung Kyun and Kang, Yisak and Yu, Xinge and Slepian, Marvin J. and Raj, Milan S. and Model, Jeffrey B. and Feng, Xue and Ghaffari, Roozbeh and Rogers, John A. and Huang, Yonggang. Relation between blood pressure and pulse wave velocity for human arteries. Proceedings of the National Academy of Sciences of the United States of America. 2018; 115(44): 11144–11149. doi: 10.1073/pnas.1814392115

9. Green, D W and Perry, R H Professional, McGraw Hill McGraw-Hill Education McGraw Hill professional Perry's Chemical Engineers' Handbook, Eighth Edition 2007

10. B. Hooper, William. The Two-k Method Predicts Head Losses in Pipe Fittings.Chemical Engineering (New York). 1981; 88: 96,98–100.

11. Olsen, O. and Schaffalitzky de Muckadell, O. B. and Cantor, P.Fat and pancreatic secretion. Scandinavian Journal of Gastroenterology. 1989; 24(1):74-80. doi: 10.3109/00365528909092242

12. Bellini, Chiara and Glass, Paul and Sitti, Metin and Di Martino, Elena S.Biaxial mechanical modeling of the small intestine. Journal of the Mechanical Behavior of Biomedical Materials. 2011; 4(8): 1727–1740. doi: 10.1016/j.jmbbm.2011.05.030

13. Villemejane, C. and Wahl, R. and Aymard, P. and Denis, S. and Michon, C. In vitro digestion of short-dough biscuits enriched in proteins and/or fibres, using a multi-compartmental and dynamic system (1): Viscosity measurement and prediction. Food Chemistry. 2015;182:55–63. doi: 10.1016/j.foodchem.2015.02.125

14. Cronin, Carmel G. and Delappe, Eithne and Lohan, Derek G. and Roche, Clare and Murphy, Joseph M. Normal small bowel wall characteristics on MR enterography. European Journal of Radiology. 2009; 75(2): 207–211. doi: 10.1016/j.ejrad.2009.04.066

15. Lema-Perez, Laura and Garcia-Tirado, Jose and Builes-Montano, Carlos and Alvarez, Hernan. Phenomenological-Based model of human stomach and its role in glucose metabolism. Journal of theoretical biology. 2019; 460: 88–100

16. Collins, P. J. and Houghton, L. A. and Read, N. W. and Chatterton, B. E. and Heddle, R. and Dent, J. and Horowitz, M. Role of the proximal and distal stomach in mixed solid and liquid meal emptying. Gut. 1991; 32(6): 615–619. doi: 10.1136/gut.32.6.615

17. Rayner, Christopher K. and Hebbard, Geoffrey S. and Horowitz, Michael Physiology of the Gastrointestinal Tract. Physiology of the Antral Pump and Gastric Emptying. 2012; 1: 959–976. doi: 10.1016/B978-0-12-382026-6.00035-X

18. Collins, P. J. and Horowitz, M. and Cook, D. J. and Harding, P. E. and Shearman, D. J. Gastric emptying in normal subjects - a reproducible technique using a single scintillation camera and computer system. Gut. 1983; 24(12): 1117–1125. doi: 10.1136/gut.24.12.1117

19. Holtzer, Alfred and Lowey, Susan. The Molecular Weight, Size and Shape of the Myosin Molecule. Journal of the American Chemical Society. 1959; 81(22): 1370–1377. doi: 10.1021/ja01515a026

20. Tortora, Gerard J. and Derrickson, Bryan H.Principles of Anatomy and Physiology. 15th ed. John Wiley & Sons. 2018: 1248.

21. Howard, P. J. and Murphy, G. M. and Dowling, R. H.Gall bladder emptying patterns in response to a normal meal in healthy subjects and patients with gall stones: Ultrasound study. Gut. 1991; 32(11):1406–1411. doi: 10.1136/gut.32.11.1406.

22. Al-Atabi, Mushtak and Ooi, R. C. and Luo, X. Y. and Chin, S. B. and Bird, N. C.Computational analysis of the flow of bile in human cystic duct. Medical Engineering and Physics. 2012; 34(8):1177–1183. doi: 10.1016/j.medengphy.2011.12.006.

23. Pandol, Stephen J. Colloquium Series on Integrated Systems Physiology: From Molecule to Function. The Exocrine Pancreas. Feb; 2011; 1(1): 1-64. doi. 10.4199/C00026ED1V01Y201102ISP014

24. Alvarez, Hernán and Peña, Miguel. Modelamiento de Sistemas de Inferencia Borrosa Tipo Takagi – Sugeno. Avances en Sistemas e Informatica. 2004 (1): 1–11

25. Keller, J. and Layer, P. Human pancreatic exocrine response to nutrients in health and disease. Gut. 2005; 54(6): 1–28. doi: 10.1136/gut.2005.065946

26. Isaksson, G. and Lundquist, I. and Ihse, I.Effect of Dietary Fiber on Pancreatic Enzyme Activity In Vitro: The Importance of Viscosity, pH, Ionic Strength, Adsorption, and Time of Incubation. Gastroenterology. 1982; 82(5): 918–924. doi: 10.1016/S0016-5085(82)80256-4

27. Teitelbaum, Ezra N. and Vaziri, Khashayar and Zettervall, Sara and Amdur, Richard L. and Orkin, Bruce A. Intraoperative small bowel length measurements and analysis of demographic predictors of increased length. Clinical Anatomy. 2013; 26(7): 827–832. doi: 10.1002/ca.22238

28. Kovacs, L. and Tuba, J. A note on the energy of activation of the amylase in various human body fluids. Canadian Journal of Biochemistry and Physiology. 1956; 34(1): 6-9. doi: 10.1139/y56-002

29. Layer, P. and Gröger, G. Fate of Pancreatic Enzymes in the Human Intestinal Lumen in Health and Pancreatic Insufficiency. Digestion. 1993; 54(2). doi: 10.1159/000201097

30. Tóth, Júlia and Simon, Zoltán and Medveczky, Péter and Gombos, Linda and Jelinek, Balázs and Szilágyi, László and Gráf, László and Málnási-Csizmadia, András. Site directed mutagenesis at position 193 of human trypsin 4 alters the rate of conformational change during activation: Role of local internal viscosity in protein dynamics. Proteins: Structure, Function, and Bioinformatics. April; 2007; 67(4): 1119–1127. doi: 10.1002/prot.21398

31. Lykidis, Athanasios and Mougios, Vassilis and Arzoglou, Pantelis. Kinetics of the two-step hydrolysis of triacylglycerol by pancreatic lipases. European Journal of Biochemistry. 1995; 230(3): 892–898. doi: 10.1111/j.1432-1033.1995.0892g.x

32. Holst, Jens Juul and Gribble, Fiona and Horowitz, Michael and Rayner, Chris K. Roles of the gut in glucose homeostasis. Diabetes Care. 2016, 39(6): 884–892

33. Allen, T. H. and Krzywicki, H. J. and Roberts, J. E. Density, fat, water and solids in freshly isolated tissues. Journal of Applied Physiology. Nov; 1959; 14(6): 1005–1008. doi: 10.1152/jappl.1959.14.6.1005

34. Helander, Herbert F. and Fändriks, Lars. Surface area of the digestive tract – revisited. Scandinavian Journal of Gastroenterology. Jun; 2014; 49(6): 681–689. doi: 10.3109/00365521.2014.898326

35. Small, P. K. and Loudon, M. A. and Hau, C. M. and Noor, N. and Campbell, F. C. Large-scale ambulatory study of postprandial jejunal motility in irritable bowel syndrome. Scandinavian Journal of Gastroenterology. 1997; 32(1): 39–47. doi: 10.3109/00365529709025061

36. Gayer, Christopher P. and Basson, Marc D.The effects of mechanical forces on intestinal physiology and pathology. Cellular Signalling. 2009; 21(8):1237–1244. doi: 10.1016/j.cellsig.2009.02.011

37. Watt, B.K. and Merrill, A.L. Handbook of the Nutritional Contents of Foods. Agriculture handbook. Dover Publications. 1975
